# The Spatiotemporal Pattern and Its Determinants of Hemorrhagic Fever With Renal Syndrome in Northeastern China: Spatiotemporal Analysis

**DOI:** 10.2196/42673

**Published:** 2023-05-18

**Authors:** Yanding Wang, Xianyu Wei, Ruizhong Jia, XingYu Peng, Xiushan Zhang, Meitao Yang, Zhiqiang Li, Jinpeng Guo, Yong Chen, Wenwu Yin, Wenyi Zhang, Yong Wang

**Affiliations:** 1 School of Public Health, China Medical University Shenyang China; 2 Chinese PLA Center for Disease Control and Prevention Beijing China; 3 School of Public Health, Anhui Medical University Hefei China; 4 Chinese Center for Disease Control and Prevention Beijing China

**Keywords:** HFRS, climate change, Northeastern China, spatiotemporal dynamic, Geodetector

## Abstract

**Background:**

Hemorrhagic fever with renal syndrome (HFRS) is a significant zoonotic disease mainly transmitted by rodents. However, the determinants of its spatiotemporal patterns in Northeast China remain unclear.

**Objective:**

This study aimed to investigate the spatiotemporal dynamics and epidemiological characteristics of HFRS and detect the meteorological effect of the HFRS epidemic in Northeastern China.

**Methods:**

The HFRS cases of Northeastern China were collected from the Chinese Center for Disease Control and Prevention, and meteorological data were collected from the National Basic Geographic Information Center. Times series analyses, wavelet analysis, Geodetector model, and SARIMA model were performed to identify the epidemiological characteristics, periodical fluctuation, and meteorological effect of HFRS in Northeastern China.

**Results:**

A total of 52,655 HFRS cases were reported in Northeastern China from 2006 to 2020, and most patients with HFRS (n=36,558, 69.43%) were aged between 30-59 years. HFRS occurred most frequently in June and November and had a significant 4- to 6-month periodicity. The explanatory power of the meteorological factors to HFRS varies from 0.15 ≤ *q* ≤ 0.01. In Heilongjiang province, mean temperature with a 4-month lag, mean ground temperature with a 4-month lag, and mean pressure with a 5-month lag had the most explanatory power on HFRS. In Liaoning province, mean temperature with a 1-month lag, mean ground temperature with a 1-month lag, and mean wind speed with a 4-month lag were found to have an effect on HFRS, but in Jilin province, the most important meteorological factors for HFRS were precipitation with a 6-month lag and maximum evaporation with a 5-month lag. The interaction analysis of meteorological factors mostly showed nonlinear enhancement. The SARIMA model predicted that 8,343 cases of HFRS are expected to occur in Northeastern China.

**Conclusions:**

HFRS showed significant inequality in epidemic and meteorological effects in Northeastern China, and eastern prefecture-level cities presented a high risk of epidemic. This study quantifies the hysteresis effects of different meteorological factors and prompts us to focus on the influence of ground temperature and precipitation on HFRS transmission in future studies, which could assist local health authorities in developing HFRS-climate surveillance, prevention, and control strategies targeting high-risk populations in China.

## Introduction

Hemorrhagic fever with renal syndrome (HFRS) is a significant zoonotic disease caused by hantavirus carried by rodent animals, such as *Apodemus agrarius* and *Rattus norvegicus* [1-3]. Hantavirus often leads to different clinical symptoms such as fever; headache; circulatory collapse with hypotension; gastrointestinal symptoms; and severe life-threatening damage, including bleeding or acute kidney injury [4-7]. Moreover, the latest research noted a 73% increased risk of lymphoma among patients with HFRS [8].

Currently, HFRS is found in more than 30 countries [9-13], such as Russia, the United States, China, Japan, and other countries, which poses a severe threat to human health and economic development. In China, the prevention and control of HFRS have been a prominent public health concern. It is estimated that about 100,000 cases of HFRS occur each year, with China being the most prevalent country [14-16], accounting for 70% to 90% of all HFRS cases and more than 200,000 cases between 2004 and 2019.

According to previous studies, Northeastern China is always the highest risk region of the HFRS epidemic in China [17-19], including Heilongjiang, Liaoning, and Jilin provinces. Since HFRS was first detected in Northeastern China in 1931, the epidemic area has gradually expanded, and cases have been reported in 31 provinces in China to date [16,20]. Although previous studies have mainly analyzed some provinces or specific cities with HFRS outbreaks in mainland China, there is also a lack of systematic evidence of the dynamics of HFRS occurrence in Northeastern China, which has the highest HFRS incidence in recent years.

In addition, the risk of HFRS is closely affected by climate factors [18,21], with temperature, wind speed, and humidity being essential drivers. Geodetector is an emerging technique in recent years and has become a reliable tool for detecting and exploiting spatial heterogeneity and determinants [22-25]. Geodetector could identify relationships between diseases and their associated factors by detecting similarities in the spatial distribution of independent and dependent variables, using the power of determinant as a metric. The traditional approach to interaction is to add the 2 factors to the regression model, but the interaction between the 2 factors is not necessarily multiplicative. Besides detecting spatial heterogeneity and the significant factors, Geodetector has the advantage of being able to detect the interaction between the 2 factors and analyze the effect of the strength, direction, and linearity or nonlinearity of the interaction in the distribution of the disease [26].

Therefore, this study combined epidemical statistics, geographic information system, and Geodetector modeling to investigate the characteristics of HFRS epidemic distribution patterns and spatiotemporal distribution and further quantify the meteorological effect of HFRS in Northeastern China, which could assist health departments in preparing targeted preventive and control strategies with a rational allocation of health resources.

## Methods

The monthly HFRS cases from Northeastern China from January 2006 to December 2020 were collected from the Chinese Center for Disease Control and Prevention (CDC). HFRS was included on the list of Class B Notifiable Diseases in China in 1950, and HFRS cases were mandatory to report to the Chinese CDC by law according to the standard and unified protocol established by the Chinese CDC. In this study, all HFRS cases were confirmed according to the diagnostic criteria for HFRS from the Ministry of Health of the People’s Republic of China [27]. The case definition for HFRS was an individual who had traveled to an HFRS endemic area or who had contact with rodent feces, saliva, and urine within 2 months before the onset of illness, with clinical manifestations such as fever, chills, hemorrhage, headache, back pain, abdominal pain, acute renal dysfunction, and hypotension. In addition, the person had to meet at least one laboratory criteria for diagnosis: a positive result for hantavirus-specific immunoglobulin M, a 4-fold rise in titers of hantavirus-specific immunoglobulin G, a positive result for hantavirus-specific ribonucleic acid by reverse transcription polymerase chain reaction in clinical specimens, or having hantavirus isolated from clinical specimens.

Monthly meteorological factors of Northeastern China were obtained from the China Meteorological Data Sharing Service System from January 1, 2006, to December 31, 2020, including mean temperature (TEM), mean pressure (PRS), mean ground temperature (GST), sunshine duration, mean relative humidity, mean wind speed, precipitation, maximum evaporation, and minimum evaporation. Demographic information was obtained from the Chinese Statistical Yearbook. The geographic data were obtained from the National Basic Geographic Information Center.

**Figure 1:**
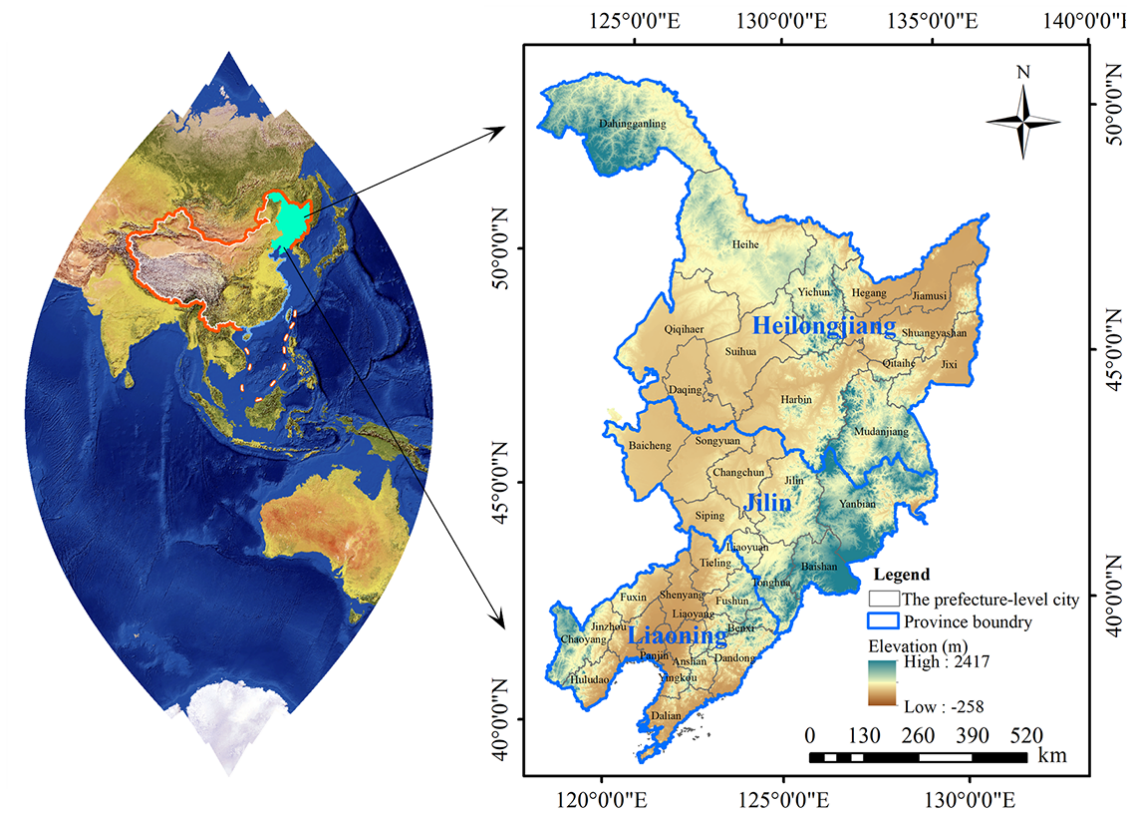
The study area in Northeastern China, which was obtained from the National Catalogue Service For Geographic Information of China [33].

Based on the previous studies, we used meteorological factors with a lag of 1-6 months [28-30] because meteorological factors showed a delayed effect on the risk of HFRS. The map is shown in Figure 1, and the mechanism route in this study is shown in Figure S1 in Multimedia Appendix 1. We analyzed the epidemiological characteristics and periodic fluctuation of HFRS with data on yearly and monthly scales. From the seasonal perspective, we classified March to May as spring, June to August as summer, September to November as autumn, and December to February as winter. Descriptive analysis of demographic characteristics of HFRS cases was mainly analyzed using Excel (version 2019; Microsoft Corp) and SPSS (version 21.0; IBM Corp). The annual incidence rate of HFRS in prefecture-level cities was calculated based on the number of new HFRS cases and the total population at the end of the year. Global spatial autocorrelation analysis, which describes the overall spatial distribution and evaluates whether the attribute has spatial aggregation [31], was used to analyze the distribution of HFRS cases using 36 cities in 3 provinces in Northeastern China. The global Moran *I* and *Z* values were computed to evaluate the clustering pattern. A positive Moran *I* value (*I*>0) means there is a positive spatial correlation and HFRS has an aggregated distribution, a Moran *I* value close to 0 means there is no autocorrelation, and a negative Moran index (*I*<0) means there is negative spatial correlation and HFRS has a discrete distribution.

Wavelet analysis was used to explore the variation of HFRS incidence periodicity [32] and detect the shift of the periodic pattern of the HFRS epidemic in Northeastern China by using Matlab (version 2016b; MathWorks Inc).

Subsequently, Geodetector modeling [22] was used to identify the governing force of a responding variable under the assumption that variable *X* is associated with variable *Y* if their spatial patterns are consistent. This study used the factor detector to detect the spatial heterogeneity of HFRS and how *X* explains the spatial patterns of HFRS using the *GD* package in R software (version 4.1.1; R Foundation for Statistical Computing). It is defined as the *q*-statistic:



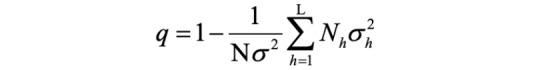

**(1)**


where variable *X* is stratified into strata *h = 1, 2, …, L*; the study area is composed of *N* units; stratum *h* is composed of *N_h_* units; *σ_h_*^2^ is the stratum variance; *σ*^2^ is the population variance; and *Y* means the HFRS cases. The *q* value indicates how the variable *X* explains 100 × *q*% of the dependent variable *Y*. For *q* [0,1], a higher value of *q* indicates a stronger spatially stratified heterogeneity (SSH) of the dependent variable *Y*. The *q* value can be used as a major factor to identify the meteorological effect of HFRS. SSH represents that the value of a certain attribute differs between different types or regions. The interaction detector could assess whether the explanatory powers of the 2 factors are enhanced, weakened, or independent of each other. The *q* values of 2 factors and their interaction were calculated. The new layer of interaction is formed by the tangent of overlay variables *X1* and *X2*. By comparing *q(X1)* and *q(X2)* with *q(X1∩X2)*, the interaction type between 2 factors can be identified: the interaction relationship between 2 factors is a bivariate enhancement when *max(q(X1), q(X2)) < q(X1∩X2) < q(X1) + q(X2)*, whereas it is a nonlinear enhancement if *q(X1∩X2) > q(X1) + q(X2)*.

Finally, the HFRS cases data in Northeastern China from 2006 to 2020 were analyzed using the seasonal autoregressive integrated moving average (SARIMA) model to forecast the HFRS cases in the next 5 years (60 months). Mean absolute error, mean absolute percentage error (MAPE), and root mean square error were used to measure the performance of the SARIMA model.

## Results

### Demographic Characteristics

A total of 52,655 cases of HFRS occurred in Northeastern China, of which 26,088, 11,386 and 15,181 cases were reported in Heilongjiang, Jilin, and Liaoning provinces, respectively, with an average of 293 new HFRS cases every month in Northeastern China from 2006 to 2020 (Table 1). Heilongjiang province has the highest incidence of HFRS in Northeastern China with 8.23 per 10,000 cases (26,088/31,710,000), followed by Jilin province with 4.75 per 10,000 cases (15,181/23,990,000) and Liaoning province with 3.57 per 10,000 cases (11,386/42,550,000).

In the analysis of population distribution characteristics of HFRS from 2006 to 2020 (Table 1), patients with HFRS were predominantly male; of the 52,655 cases, 40,664 (77.23%) were male and 11,991 (22.77%) were female, with a sex ratio of 3.4:1. Most patients with HFRS (n=36,558, 69.43%) were aged between 30-59 years, of which 38.55% (14,092/36,558) were in the 40-49 years age group.

**Table 1:** The characteristics of hemorrhagic fever with renal syndrome cases in Northeast China from 2006 to 2020.

		Heilongjiang province		Jilin province		Liaoning province		Northeastern China		
		Male, n	Female, n	Male, n	Female, n	Male, n	Female, n	Male, n	Female, n	Total, n
**Age group (years)**										
	0-9	25	21	23	14	15	24	63	59	122
	10-19	749	174	354	91	446	156	1549	421	1970
	20-29	2945	591	1253	203	1370	333	5568	1127	6695
	30-39	4831	930	2147	371	2162	604	9140	1905	11,045
	40-49	5637	1395	2541	581	2937	1001	11,115	2977	14,092
	50-59	3976	1449	1808	611	2435	1142	8219	3202	11,421
	60-69	1811	751	749	319	1258	646	3818	1716	5534
	70-79	483	236	182	93	368	183	1033	512	1545
	≥80	61	23	30	16	68	33	159	72	231
Total		20,518	5570	9087	2299	11,059	4122	40,664	11,991	52,655

### Temporal Distribution of HFRS

The analysis of the number of HFRS incidences over 15 years shows that the number of HFRS cases in Northeastern China generally showed a decreasing trend over years, mainly with a rapid decrease from 2006 to 2010. An increasing trend was demonstrated from 2011 to 2014, followed by a fluctuation reduction between 2011 and 2016. There was a rising trend between 2016 and 2018 and a decreasing trend between 2019 and 2020.

As for the monthly characteristics, this study also showed that HFRS cases were seasonal, including 2 HFRS epidemic peaks: summer and winter peaks. The epidemic months of Northeastern China were different in the 3 provinces.

**Figure 2:**
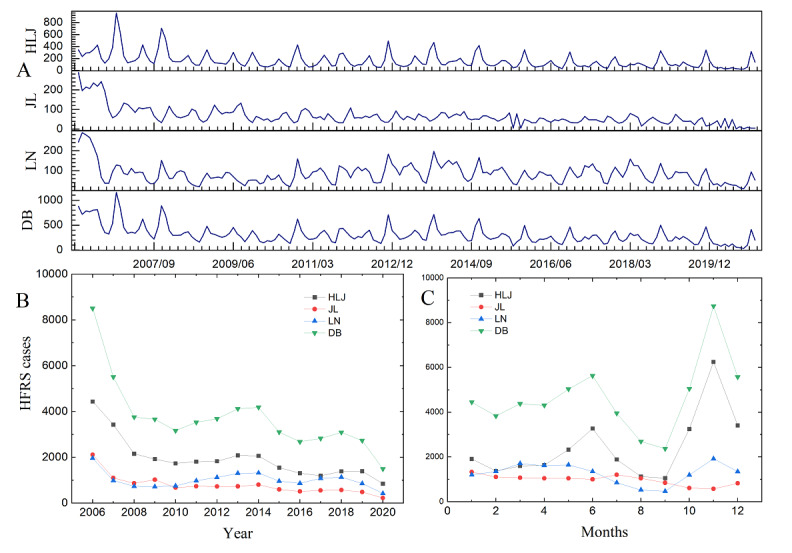
Yearly and monthly variations of HFRS in Northeastern China from 2006 to 2020. (A) Monthly time series change of HFRS cases from January 2006 to December 2020. (B) Yearly variation of HFRS cases in the 3 provinces and Northeastern China. (C) Seasonal variation of the HFRS cases in the 3 provinces and Northeastern China. DB: Northeastern China; HFRS: hemorrhagic fever with renal syndrome; HLJ: Heilongjiang province; JL: Jilin province; LN: Liaoning province.

In November, Northeastern China had the highest HFRS incidence with 0.88 per 10,000 cases (8676/98,250,000), followed by a lower incidence in June with 0.59 per 10,000 cases (5818/98,250,000). Specifically, Heilongjiang province had a prominent winter peak of HFRS from 2006 to 2020, and HFRS cases spiked in November, accounting for 1.75 per 10,000 cases (5539/31,710,000) in Heilongjiang province in November. However, the number of HFRS cases in Liaoning province peaked in late autumn, with cases concentrated in November with 0.45 per 10,000 cases (1921/42,550,000) in Liaoning province, which differed slightly from the second incidence peak of 0.40 per 10,000 cases (1706/42,550,000) in March. The monthly and seasonal variation trends of HFRS cases in the 3 provinces of Northeastern China are shown in Figure 2.

### Wavelet Analysis

**Figure 3:**
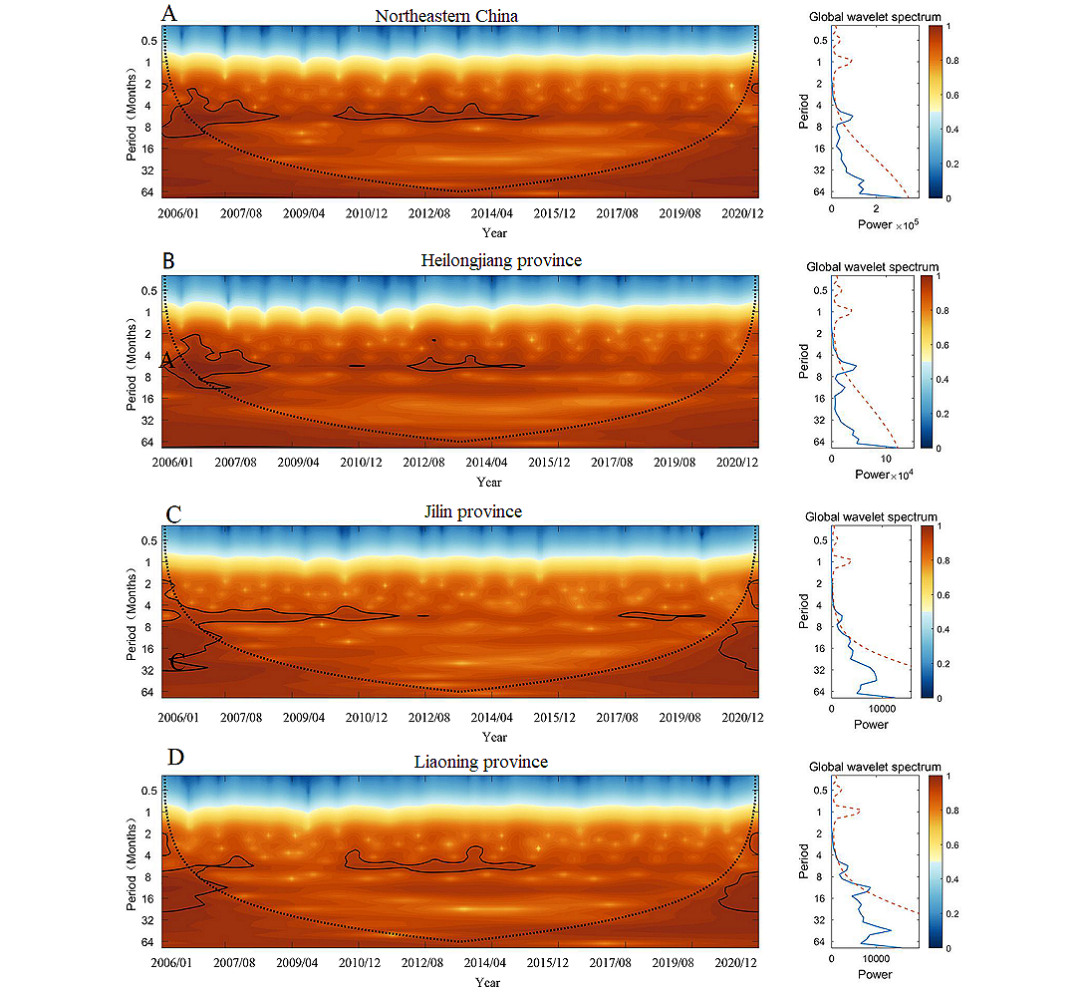
Wavelet power spectrum of monthly HFRS cases in Northeastern China. A mean wavelet coherency spectrum and variance of HFRS wavelet coherency spectrum in the northeast region of China between January 2006 and December 2020. The black line represents the influence cone that delimits the region that is totally not influenced by edge effects. (A) Northeastern China, (B) Heilongjiang province, (C) Jilin province, and (D) Liaoning province. Blue line: global average value of the wavelet spectrum; red dot line: global average value of the spectrum with 95% confidence. HFRS: hemorrhagic fever with renal syndrome.

Wavelet analysis was performed on HFRS cases in Northeastern China for a total of 180 months from 2006 to 2020, and the main periodicities and variation characteristics of the 3 provinces were identified. The results are shown in Figure 3.

Wavelet power spectra detected a significant 4- to 6-month periodic mode for HFRS in Northeastern China from 2006 to 2020 (Figure 3A), which was characterized by a strong periodical fluctuation of 2 to 8 months from 2006 to 2009, while a dominant shift period of 4 to 6 months existed from 2010 to 2015. In Heilongjiang province (Figure 3B), a 2- to 8-month periodicity from 2006 to 2009 and an approximately 4- to 6-month periodicity from 2014 to 2015 were revealed. The HFRS periodicity in Jilin province was 4 to 6 months, mainly concentrated from 2007 to 2014 and from 2017 to 2019 (Figure 3C). In Liaoning province, the periodicity was primarily 4 to 6 months from 2010 to 2015 (Figure 3D).

### Spatial Distribution and Cluster

**Figure 4:**
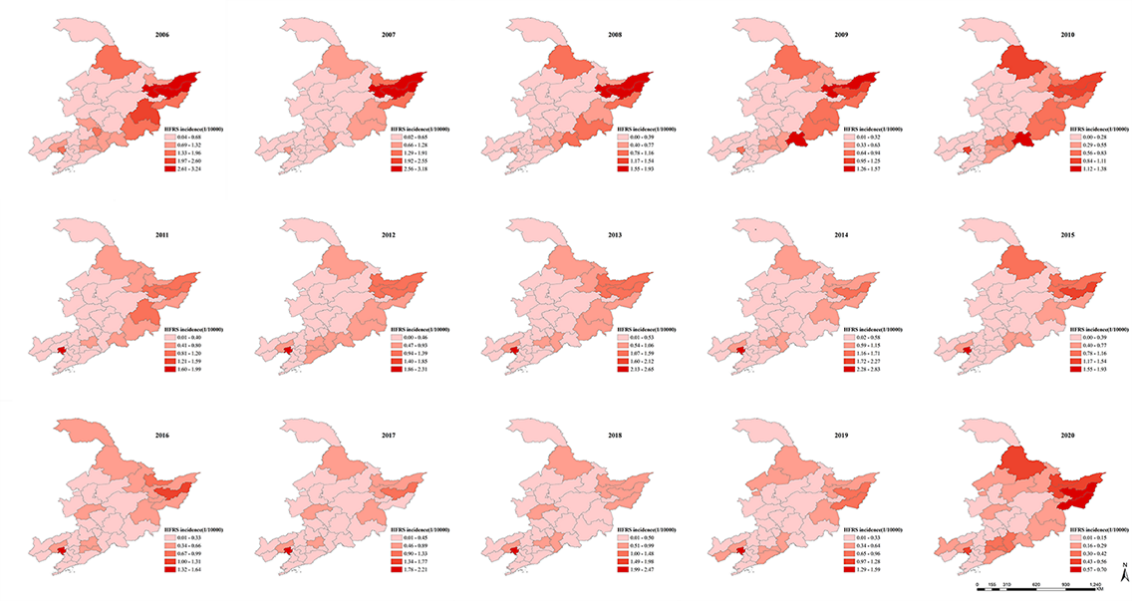
Yearly distribution of hemorrhagic fever with renal syndrome (HFRS) incidence in Northeastern China, from 2006 to 2020. For a higher-resolution version of this figure, see Multimedia Appendix 1.

The annual incidence rate of HFRS was unequally distributed among prefecture-level cities in Northeastern China during the 15 years and displayed a decreasing trend by year (Figure 4). Between 2006 and 2010, the areas with high incidence rates of HFRS were mainly concentrated in the eastern and northern parts of Jiamusi City and Shuangyashan City in Heilongjiang province. In contrast, between 2011 and 2015, the number of HFRS cases in cities in Northeastern China gradually decreased. The city with the highest incidence shifted from Shuangyashan City, Jiamusi City, and Hegang City in Heilongjiang province to Panjin City in Liaoning province. Although HFRS cases were still mainly concentrated in eastern Heilongjiang province, the number of patients in the 2 main endemic areas of Jiamusi City and Shuangyashan City decreased substantially, and the distribution range did not expand. Between 2016 and 2020, the trend of HFRS occurrence decreased from east to west. The cities with the annual highest HFRS incidence were Shuangyashan City and Jixi City in the east. The east coastal cities in Northeastern China’s southern part had also become one of the high-risk regions of HFRS, similar to the distribution between 2006 and 2010. Spatial autocorrelation analysis of HFRS incidence for 15 years showed a spatially positive correlation. Moran index fluctuated from 0.02 to 0.43 from 2006 to 2020. For most of the time, HFRS showed a spatially positive correlation with significant spatial aggregation rather than random distribution (Table S1 in Multimedia Appendix 1). The largest Moran index and the strongest aggregation effect was in 2007 during the 15 years (*I*=0.43; *Z*=4.56). There was a positive spatial correlation and aggregated distribution of HFRS prevalence from 2005 to 2010 and in 2016 and 2020, and its overall trend was weakening.

### Geodetector Modeling

HFRS in Northeastern China was characterized by significant SSH. The results of this study showed that the dominant meteorological factors influencing the incidence of HFRS in different regions in Northeastern China had significant differences (all *P*<.001; Table S1 in Multimedia Appendix 2). In Heilongjiang province, the meteorological factors with the greatest influence on HFRS were TEM with a 4-month lag (15.49%), GST with a 4-month lag (14.65%), and PRS with a 5-month lag (7.35%). In Liaoning province, TEM with a 1-month lag (5.79%), GST with a 1-month lag (5.35%), and mean wind speed with a 4-month lag (4.94%) were the top 3 determinants. However, the meteorological factors with the greatest influence on HFRS in Jilin province were precipitation with a 6-month lag (5.52%), maximum evaporation with a 5-month lag (5.36%), and PRS (5.26%). GST with a 1-month lag (4.18%), precipitation with a 3-month lag (3.54%), and TEM with a 4-month lag (3.31%) also had a significant effect on the prevalence of HFRS in Northeastern China (all *P*<.001), as shown in Table S1 in Multimedia Appendix 2.

The effect of any 2 combination of different meteorological factors on HFRS was also quantified simultaneously (Tables S2-5 in Multimedia Appendix 2). The effect of individual factors on the onset of HFRS was enhanced after combining 2 factors compared to the effect of the factors alone. In Heilongjiang province, the *q* value of PRS with a 5-month lag and GST with a 4-month lag was 0.2680. This indicates the strongest interaction of PRS with a 5-month lag and GST with a 4-month lag could explain 26.80% of the HFRS incidence, which also means that the 2 factors together had a higher interaction than their respective independent effects (Table S5 Multimedia Appendix 2).

In Jilin province, the *q* value of precipitation with a 6-month lag and PRS was 0.1289, and the explanatory power of the 2 interactions was greater than the sum of the explanatory power of their independent effects, indicating a nonlinear enhancement effect (Table S3 in Multimedia Appendix 2). In Liaoning province, mean relative humidity with a 1-month lag and TEM with a 1-month lag have an explanatory power of 10.90% for the HFRS epidemic (Table S4 in Multimedia Appendix 2).

### Time Series Forecasting of HFRS

By constructing the SARIMA model (4, 1, 3)(1, 0, 1)[4], a global trend of fluctuating decrease in HFRS cases was predicted in Northeastern China over the next 5 years. The results showed the training accuracy of the SARIMA model (MAPE=0.17) and the summary performance with the HFRS data set (MAPE=0.23; Table S6 in Multimedia Appendix 2). The model predicted that 8343 cases of HFRS are expected to occur in Northeastern China, with a fluctuation range of 4 to 250 cases per month.

## Discussion

### Principal Findings

This long-term analysis from Northeastern China is the first comprehensive work to interpret the spatiotemporal distribution and different explanatory power of various meteorological variables for HFRS from 2006 to 2020, providing essential clues to understanding how HFRS developed.

### Comparison With Prior Work

From 2006 to 2020, the epidemic situation of HFRS in Northeastern China was complex and severe, with an overall fluctuating downward trend. The epidemic was widespread in Northeastern China, with reports from 36 cities within the range. However, the risk of HFRS incidence varied widely among prefectures. The distribution of prefecture-level cities in Northeastern China is shown in Figure 1. This decreasing trend is consistent with previous studies [34,35] and primarily due to the implementation of infectious disease prevention and control measures of the Chinese government. With the development of the economy, improvement of medical conditions, and promotion and application of vaccines, the incidence of HFRS in China has been effectively controlled, and the overall incidence rate has decreased significantly [36,37]. Since the Chinese government’s implementation of the HFRS immunization program and approximately 2 million doses of HFRS vaccine having been administered annually in China, the distribution and incidence of HFRS infection have changed significantly [38,39]. The evidence revealed a negative correlation between HFRS incidence and vaccination adherence and that vaccination-induced progressive reduction of viral infection in susceptible populations prevents further epidemics and has a notable effect on HFRS prevention and control.

High prevalence of HFRS is found in male individuals in the 30-59 years age group. There are significant differences in the spatial and temporal distribution of HFRS cases in Northeastern China. The eastern and southern prefecture-level cities were the high-risk clusters of HFRS epidemic in Northeastern China, primarily in Heilongjiang province, such as Qitaihe city, Heihe city, and Harbin city. This may be due to the fact that middle-aged male individuals have more exposure to host animals than other populations when they work in endemic areas [40,41]; it also suggests that middle-aged male individuals are a high-risk group for HFRS exposure. In addition, the distribution and number of HFRS host animal populations are influenced by environmental conditions such as temperature and geography, resulting in different distribution and prevalence intensities of HFRS in the different prefecture-level cities [42-44].

The study by He et al [45] suggest that the influence of climate on HFRS becomes increasingly sensitive over time, and it revealed an important clue that the geographic factors with the most significant impact on climate-HFRS association include latitude, distance, and longitude from the coastline, grassland, and woodland. In this study, Heilongjiang province is a prime HFRS epidemic area with a higher incidence of HFRS than the other 2 provinces. The dynamics of HFRS incidence may be due to unique local natural climatic, geographic, and socioeconomic factors. Heilongjiang province is a low mountainous region with high southeast and low northwest topography, located in the midlatitude zone with a cold-temperate continental monsoon climate. Since high-risk HFRS cities are located at the center of the eastern part of Heilongjiang province, their convenient transportation, frequent trade, and tourism with the surrounding areas may, to some extent, contribute to the spread of HFRS. In addition, the land types in the eastern cities are mainly forested and arable land; for example, the arable land area in Qitaihe City accounts for about 31.3% of the total land area of the city and forested land accounts for about 52.8% [46]. Land types are related to the risk of HFRS, which implies that with the development of woodland and farmland, new habitats for hantavirus hosts may also be more available, increasing host density in areas of human activity and further enhancing the risk of HFRS transmission.

The re-emergence of Jiamusi City and Shuangyashan City as hot spots of HFRS in 2020 also suggests that we should focus on strengthening the prevention and control of the epidemic in this region. In addition, the predictions of the SARIMA model suggest that we are still at risk of an epidemic of HFRS in the next 5 years. This information helps health officials better understand the epidemiological patterns of HFRS and encourages them to adapt monitoring, prevention, and control strategies to the HFRS epidemic’s periodicity and seasonal fluctuations in Northeastern China.

Additionally, in this study, the seasonality of HFRS was related to the climate condition, with 2 peak incidences per year—summer and winter—and the peak incidence in winter is higher than that in summer in most years. This finding suggests that the influences of meteorological factors could partly explain seasonal variations of HFRS. This characteristic is consistent with previous results [47], which showed that the spatiotemporal dynamics of HFRS cases in Jiangxi province, that is, the HFRS epidemic, has a bimodal seasonal pattern with the primary peak occurring in winter and the secondary peak in early summer. This is because the difference in HFRS incidence between winter and summer is accompanied by the difference in climate between seasons, indicating that changes in meteorological factors play an important role in the dynamics of HFRS [43,48]. Studies have shown seasonal peaks in the HFRS outbreak in China [17,49], and in this study, HFRS risk was much higher in spring in Liaoning and Jilin provinces, and there was a higher peak in Heilongjiang province in winter. The changes in climatic factors are the significant driving force of HFRS and are an important sensitive reason for the occurrence of HFRS [20,45]. In summer and winter, the obvious changes in temperature and precipitation could affect aerosols and crop growth, which provide the conditions for rodent activities [36]; for example, rats may congregate in residential areas during a cold winter to live, increasing the density of rats and their contact with people, which may accelerate the spread of hantavirus [19,43,50].

### Possible Implications for Public Health

This study highlights the role of GST, whose separate factor effects were detected to be larger than the effect of temperature and rainfall (Heilongjiang province). This is an outstanding finding of this paper, as few studies have included this indicator in the survey of HFRS. The level of GST is closely related to the growth and development of crops, decomposition of fertilizers, and accumulation of organic matter and is an important environmental factor in plant growth [51]. GST also plays a highly crucial role in microclimate formation [52,53], so it can be hypothesized that soil temperature further influences the survival of host animals by affecting the growth of vegetation and soil microbes, which ultimately leads to changes in host-human transmission of the hantavirus. Furthermore, the interaction of meteorological factors on HFRS was analyzed by the Geodetector, and the interaction of the 2 factors was found to be significantly higher than that of the factors alone. Most of the other meteorological factors interacting with the GST factor also showed enhanced effects, further illustrating the importance of the GST factor.

### Limitations

The results of this study also have some limitations. The spatial scale of this study was based on the monthly HFRS case characteristics at the prefecture-level cities in Northeastern China, which may miss some smaller-scale characteristics of HFRS incidence at the county level. Moreover, the HFRS data were obtained from the National Passive Surveillance System, and some unreported data could not be collected. In addition, the current research focused on the temporal and spatial dynamics of HFRS in Northeastern China and has yet to include the factors influencing the disease, such as rodent density, socioeconomic level, and so on. In future studies, more factors will be collected to analyze the relationship between HFRS and environmental-social factors.

### Conclusion

Climate factor fluctuations significantly shape the spatiotemporal pattern of HFRS, especially the ground temperature and precipitation factors. Prevention and management should be further focused on high-risk provinces and cities, such as the eastern part of Northeastern China. The health department should make comprehensive prevention and control policies and take measures in advance to prevent HFRS outbreaks in summer and winter. This study will enhance the understanding of meteorological effects of HFRS transmission in Northeastern China over the last 15 years and is helpful to the high-risk region for the scientific adoption of prevention and control strategies of zoonotic disease.

## Appendix

Multimedia Appendix 1 Spatial autocorrelation results, mechanism route, and yearly distribution of hemorrhagic fever with renal syndrome (HFRS) incidence.

Multimedia Appendix 2 Factor detector, interaction detector, and seasonal autoregressive integrated moving average (SARIMA) model results.

## Abbreviations

CDC: Center for Disease Control and Prevention
GST: mean ground temperature
HFRS: hemorrhagic fever with renal syndrome
MAPE: mean absolute percentage error
PRS: mean pressure
SARIMA: seasonal autoregressive integrated moving average
SSH: spatially stratified heterogeneity
TEM: mean temperature

## References

[1] Schmaljohn CS, Hasty SE, Harrison SA, Dalrymple JM (1983). Characterization of Hantaan virions, the prototype virus of hemorrhagic fever with renal syndrome. J Infect Dis.

[2] Jiang H, Zheng X, Wang L, Du H, Wang P, Bai X (2017). Hantavirus infection: a global zoonotic challenge. Virol Sin.

[3] Sundberg E, Hultdin J, Nilsson S, Ahlm C (2011). Evidence of disseminated intravascular coagulation in a hemorrhagic fever with renal syndrome-scoring models and severe illness. PLoS One.

[4] Jiang H, Du H, Wang LM, Wang PZ, Bai XF (2016). Hemorrhagic fever with renal syndrome: pathogenesis and clinical picture. Front Cell Infect Microbiol.

[5] Zhu Y, Chen Y, Zhu Y, Liu P, Zeng H, Lu N (2013). A retrospective study of acute pancreatitis in patients with hemorrhagic fever with renal syndrome. BMC Gastroenterol.

[6] Hong YM, Moon JC, Yang HC, Kang KP, Kim W, Park SK, Lee S (2012). Hemorrhagic fever with renal syndrome and coexisting hantavirus pulmonary syndrome. Kidney Res Clin Pract.

[7] Huong VTQ, Yoshimatsu K, Luan VD, Tuan Le Van, Nhi L, Arikawa J, Nguyen TMN (2010). Hemorrhagic fever with renal syndrome, Vietnam. Emerg Infect Dis.

[8] Yi YJ, Kang M, Kim WK, Huh K, Klingström J, Song JW, Jung J (2021). Association between haemorrhagic fever with renal syndrome and cancers. Int J Infect Dis.

[9] Klingström Jonas, Granath F, Ekbom A, Björkström Niklas K, Ljunggren H (2014). Increased risk for lymphoma following hemorrhagic fever with renal syndrome. Clin Infect Dis.

[10] Zhang Y, Zhang F, Wang Jian-Bo, Zhao Zhi-Wei, Li Ming-Hui, Chen Hua-Xin, Zou Yang, Plyusnin Alexander (2009). Hantaviruses in rodents and humans, Inner Mongolia Autonomous Region, China. Emerg Infect Dis.

[11] Tkachenko EA, Ishmukhametov AA, Dzagurova TK, Bernshtein AD, Morozov VG, Siniugina AA, Kurashova SS, Balkina AS, Tkachenko PE, Kruger DH, Klempa B (2019). Hemorrhagic fever with renal syndrome, Russia. Emerg Infect Dis.

[12] Schneider F, Vidal L, Auvray C, Khider Y, Graesslin O (2009). The first French hemorrhagic fever with renal syndrome in pregnant woman. Article in French. J Gynecol Obstet Biol Reprod (Paris).

[13] Morita C, Sugiyama K, Matsuura Y, Kitamura T, Komatsu T, Akao Y, Jitsukawa W, Sakakibara H (1983). Detection of antibody against hemorrhagic fever with renal syndrome (HFRS) virus in sera of house rats captured in port areas of Japan. Jpn J Med Sci Biol.

[14] Zhang YZ, Zou Y, Fu ZF, Plyusnin A (2010). Hantavirus infections in humans and animals, China. Emerg Infect Dis.

[15] Zou LX, Sun L (2020). Analysis of hemorrhagic fever with renal syndrome using wavelet tools in mainland China, 2004-2019. Front Public Health.

[16] Zhang WY, Wang LY, Liu YX, Yin WW, Hu WB, Magalhaes RJS, Ding F, Sun HL, Zhou H, Li SL, Haque U, Tong S, Glass GE, Bi P, Clements ACA, Liu Q, Li C (2014). Spatiotemporal transmission dynamics of hemorrhagic fever with renal syndrome in China, 2005-2012. PLoS Negl Trop Dis.

[17] Shang C, Sun Y, Yin Q, Huang X, Liu X, Zhang Q, Mao L, Li C, Li A, Wang Qin, Sun Lina, Liang Mifang, Wang Shiwen, Li Dexin, Li Jiandong (2020). Hemorrhagic fever with renal syndrome - Liaoning Province, China, 1999-2018. China CDC Wkly.

[18] Li CP, Cui Z, Li SL, Magalhaes RJS, Wang B, Zhang C, Sun H, Li C, Huang L, Ma Jun, Zhang Wen-Yi (2013). Association between hemorrhagic fever with renal syndrome epidemic and climate factors in Heilongjiang Province, China. Am J Trop Med Hyg.

[19] He J, Christakos G, Wu J, Jankowski P, Langousis A, Wang Y, Yin W, Zhang W (2019). Probabilistic logic analysis of the highly heterogeneous spatiotemporal HFRS incidence distribution in Heilongjiang province (China) during 2005-2013. PLoS Negl Trop Dis.

[20] Wu W, Guo JQ, Yin Z, Wang PH, Zhou BS (2009). GIS-based spatial, temporal, and space-time analysis of haemorrhagic fever with renal syndrome. Epidemiol Infect.

[21] Li S, Ren H, Hu W, Lu L, Xu X, Zhuang D, Liu Q (2014). Spatiotemporal heterogeneity analysis of hemorrhagic fever with renal syndrome in China using geographically weighted regression models. Int J Environ Res Public Health.

[22] Wang JF, Zhang TL, Fu BJ (2016). A measure of spatial stratified heterogeneity. Ecological Indicators.

[23] Zeng W, Wan X, Lei M, Gu G, Chen T (2022). Influencing factors and prediction of arsenic concentration in Pteris vittata: a combination of geodetector and empirical models. Environ Pollut.

[24] Chen Y, Zhou Y, Zhang H, Wang C, Wang X, NixiaCiren, GesangDeji (2022). Spatiotemporal variations of surface ozone and its influencing factors across Tibet: a Geodetector-based study. Sci Total Environ.

[25] Wang Z, Li X, Zhao H (2022). Identifying spatial influence of urban elements on road-deposited sediment and the associated phosphorus by coupling Geodetector and Bayesian Networks. J Environ Manage.

[26] Song Y, Wang J, Ge Y, Xu C (2020). An optimal parameters-based geographical detector model enhances geographic characteristics of explanatory variables for spatial heterogeneity analysis: cases with different types of spatial data. GIScience & Remote Sensing.

[27] Ministry of Health (1998). Handbook of Epidemic Hemorrhagic Fever Prevention and Control.

[28] Luo Y, Lv H, Yan H, Zhu C, Ai L, Li W, Yi J, Zhang L, Tan W (2022). Meteorological change and hemorrhagic fever with renal syndrome epidemic in China, 2004-2018. Sci Rep.

[29] Xu Q, Li R, Rutherford S, Luo C, Liu Y, Wang Z, Li X (2018). Using a distributed lag non-linear model to identify impact of temperature variables on haemorrhagic fever with renal syndrome in Shandong Province. Epidemiol Infect.

[30] Wang Y, Wei X, Xiao X, Yin W, He J, Ren Z, Li Z, Yang M, Tong S, Guo Y, Zhang W, Wang Y (2022). Climate and socio-economic factors drive the spatio-temporal dynamics of HFRS in Northeastern China. One Health.

[31] Moran PAP (2018). The interpretation of statistical maps. J R Stat Soc Series B Stat Methodol.

[32] Cazelles B, Chavez M, Magny GCD, Guégan Jean-Francois, Hales S (2007). Time-dependent spectral analysis of epidemiological time-series with wavelets. J R Soc Interface.

[33] National Catalogue Service For Geographic Information of China.

[34] Sun L, Zou LX (2018). Spatiotemporal analysis and forecasting model of hemorrhagic fever with renal syndrome in mainland China. Epidemiol Infect.

[35] Liu Q, Liu X, Jiang B, Yang W (2011). Forecasting incidence of hemorrhagic fever with renal syndrome in China using ARIMA model. BMC Infect Dis.

[36] Tian H, Hu S, Cazelles B, Chowell G, Gao L, Laine M, Li Y, Yang H, Li Y, Yang Q, Tong X, Huang R, Bjornstad ON, Xiao H, Stenseth NC (2018). Urbanization prolongs hantavirus epidemics in cities. Proc Natl Acad Sci U S A.

[37] Brocato RL, Hooper JW (2019). Progress on the prevention and treatment of hantavirus disease. Viruses.

[38] Wu W, Guo J, An S, Guan P, Ren Y, Xia L, Zhou B (2015). Comparison of two hybrid models for forecasting the incidence of hemorrhagic fever with renal syndrome in Jiangsu Province, China. PLoS One.

[39] Li Z, Zeng H, Wang Y, Zhang Y, Cheng L, Zhang F, Lei Y, Jin B, Ma Y, Chen L (2017). The assessment of Hantaan virus-specific antibody responses after the immunization program for hemorrhagic fever with renal syndrome in northwest China. Hum Vaccin Immunother.

[40] Turčinov D, Puljiz I, Markotić A, Kuzman I, Begovac J (2013). Clinical and laboratory findings in patients with oliguric and non-oliguric hantavirus haemorrhagic fever with renal syndrome: an analysis of 128 patients. Clin Microbiol Infect.

[41] Koch J, Brockmann S, Winter C, Kimmig P, Stark K (2007). Significant increase of hantavirus infections in Germany since the beginning of 2007. Euro Surveill.

[42] Bai XH, Peng C, Jiang T, Hu ZM, Huang DS, Guan P (2019). Distribution of geographical scale, data aggregation unit and period in the correlation analysis between temperature and incidence of HFRS in mainland China: a systematic review of 27 ecological studies. PLoS Negl Trop Dis.

[43] Tian H, Stenseth NC (2019). The ecological dynamics of hantavirus diseases: from environmental variability to disease prevention largely based on data from China. PLoS Negl Trop Dis.

[44] Lin H, Liu Q, Guo J, Zhang J, Wang J, Chen H (2007). Analysis of the geographic distribution of HFRS in Liaoning Province between 2000 and 2005. BMC Public Health.

[45] He J, Christakos G, Wu J, Cazelles B, Qian Q, Mu D, Wang Y, Yin W, Zhang W (2018). Spatiotemporal variation of the association between climate dynamics and HFRS outbreaks in Eastern China during 2005-2016 and its geographic determinants. PLoS Negl Trop Dis.

[46] Government of Qitaihe City, China.

[47] Yang S, Gao Y, Liu X, Liu X, Liu Y, Metelmann S, Yuan C, Yue Y, Chen S, Liu Q (2020). Spatiotemporal dynamics of hemorrhagic fever with renal syndrome in Jiangxi province, China. Sci Rep.

[48] Zhang W, Guo W, Fang L, Li C, Bi P, Glass GE, Jiang J, Sun S, Qian Q, Liu W, Yan L, Yang H, Tong S, Cao W (2010). Climate variability and hemorrhagic fever with renal syndrome transmission in Northeastern China. Environ Health Perspect.

[49] Zhang S, Wang S, Yin W, Liang M, Li J, Zhang Q, Feng Z, Li D (2014). Epidemic characteristics of hemorrhagic fever with renal syndrome in China, 2006-2012. BMC Infect Dis.

[50] Wu G, Xia Z, Wang F, Wu J, Cheng D, Chen X, Liu H, Du Z (2020). Investigation on risk factors of haemorrhagic fever with renal syndrome (HFRS) in Xuancheng City in Anhui Province, mainland China. Epidemiol Infect.

[51] Olivera Viciedo D, de Mello Prado R, Martinez CA, Habermann E, de Cássia Piccolo Marisa, Calero Hurtado A, Barreto RF, Peña Calzada Kolima (2021). Changes in soil water availability and air-temperature impact biomass allocation and C:N:P stoichiometry in different organs of Stylosanthes capitata Vogel. J Environ Manage.

[52] Dacal M, Bradford MA, Plaza C, Maestre FT, García-Palacios Pablo (2019). Soil microbial respiration adapts to ambient temperature in global drylands. Nat Ecol Evol.

[53] Moinet GYK, Dhami MK, Hunt JE, Podolyan A, Liáng Liyĭn L, Schipper LA, Whitehead D, Nuñez Jonathan, Nascente A, Millard P (2021). Soil microbial sensitivity to temperature remains unchanged despite community compositional shifts along geothermal gradients. Glob Chang Biol.

