# Supplementary material for: The Spatiotemporal Pattern and Its Determinants of Hemorrhagic Fever With Renal Syndrome in Northeastern China: Spatiotemporal Analysis
Source: JMIR Public Health Surveill. 2023 May 18;9:e42673. doi: 10.2196/42673 (PMC10236282; doi:10.2196/42673)
Supplement: Multimedia Appendix 1 [file publichealth_v9i1e42673_app1.docx]

| **Table S1. Spatial autocorrelation results of monthly HFRS cases in Northeastern China, China, 2006–2020.** | | | |
| --- | --- | --- | --- |
| Year | Moran’s I | Z-score | P-value |
| 2006 | 0.1988 | 2.2208 | ＜0.05 |
| 2007 | 0.4277 | 4.5553 | ＜0.01 |
| 2008 | 0.3662 | 3.7753 | ＜0.01 |
| 2009 | 0.3129 | 3.0761 | ＜0.01 |
| 2010 | 0.2721 | 2.7970 | ＜0.01 |
| 2011 | 0.1160 | 1.5301 | 0.07 |
| 2012 | 0.115 | 1.5145 | 0.07 |
| 2013 | 0.1113 | 1.5450 | 0.07 |
| 2014 | 0.0744 | 1.1340 | 0.13 |
| 2015 | 0.1108 | 1.4594 | 0.07 |
| 2016 | 0.1319 | 1.7726 | ＜0.05 |
| 2017 | 0.0413 | 0.9017 | 0.17 |
| 2018 | 0.0139 | 0.5970 | 0.26 |
| 2019 | 0.0862 | 1.2386 | 0.11 |
| 2020 | 0.2241 | 2.3706 | ＜0.01 |

**
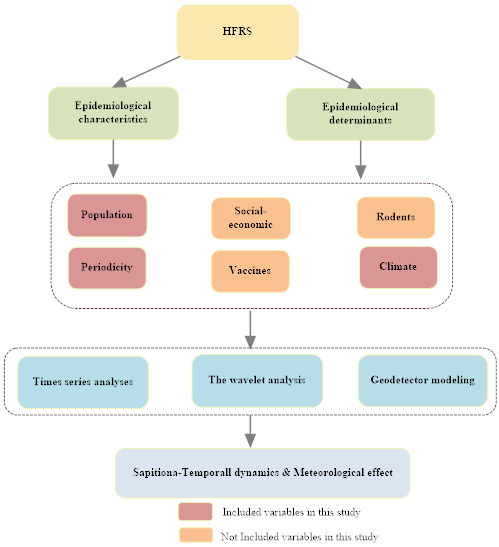
**

**Figure S1.** The mechanism route of HFRS in Northeastern China

**
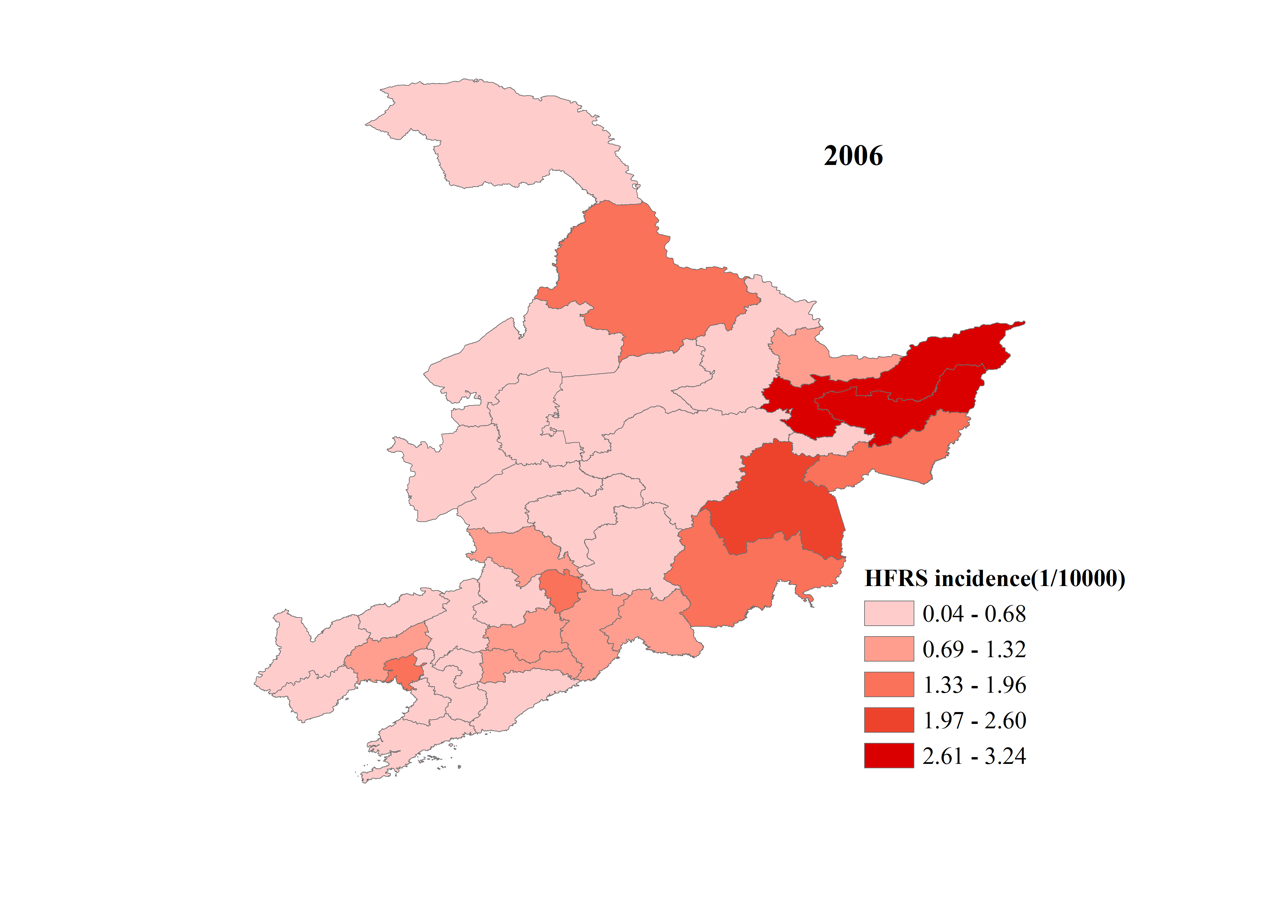
**

**Figure S2.** Yearly distribution of HFRS incidence in Northeastern China, 2006.

**
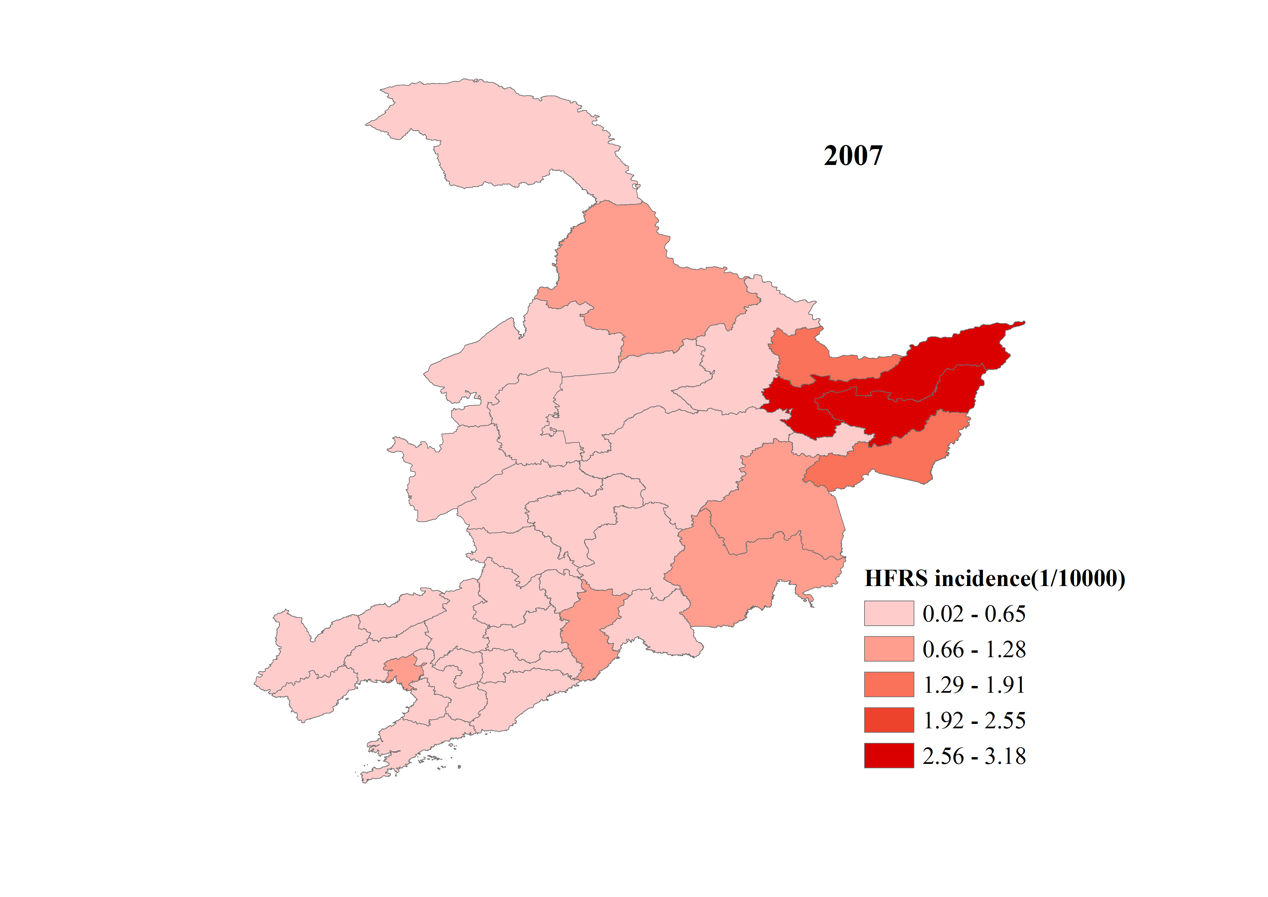
**

**Figure S3.** Yearly distribution of HFRS incidence in Northeastern China, 2007.


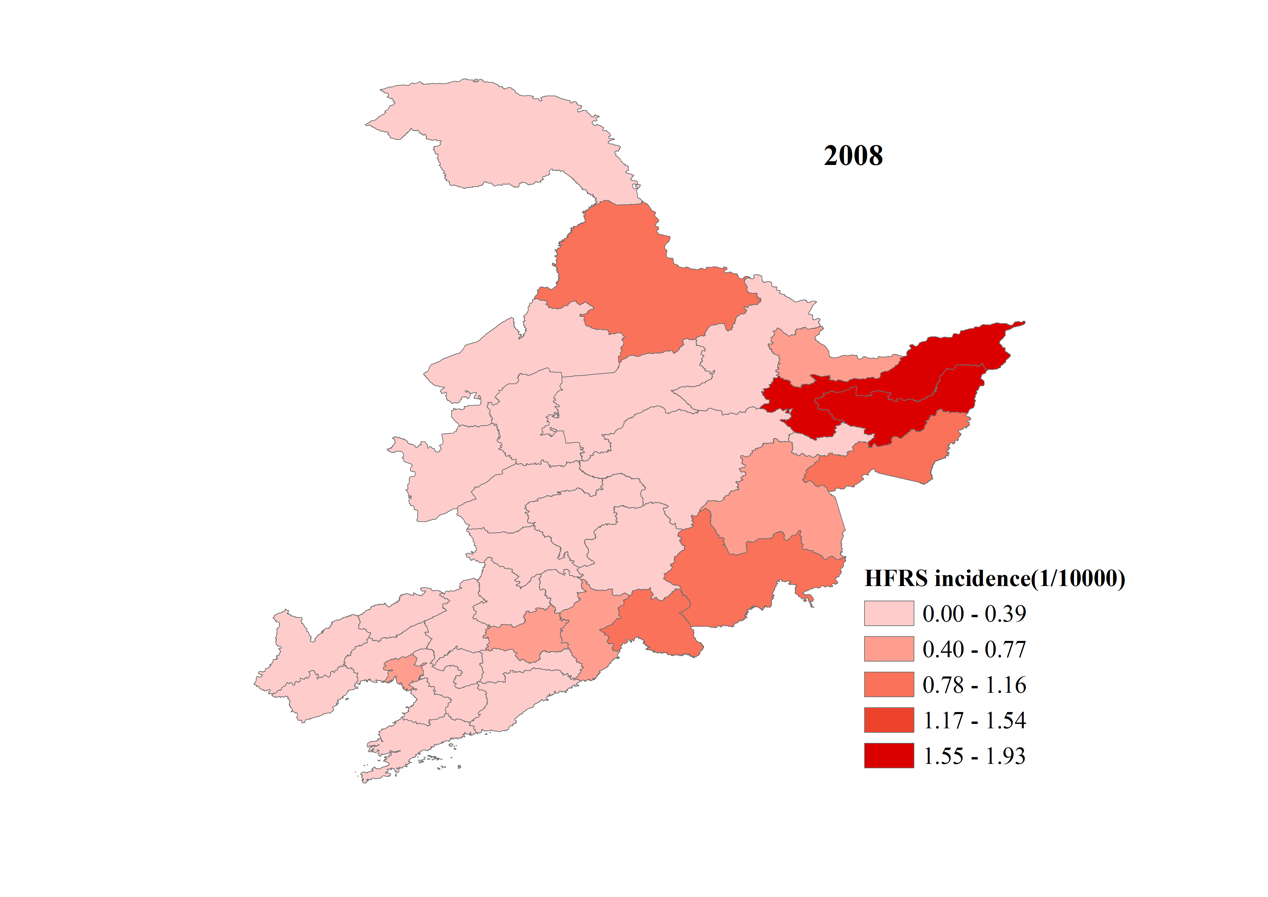


**Figure S4.** Yearly distribution of HFRS incidence in Northeastern China, 2008.


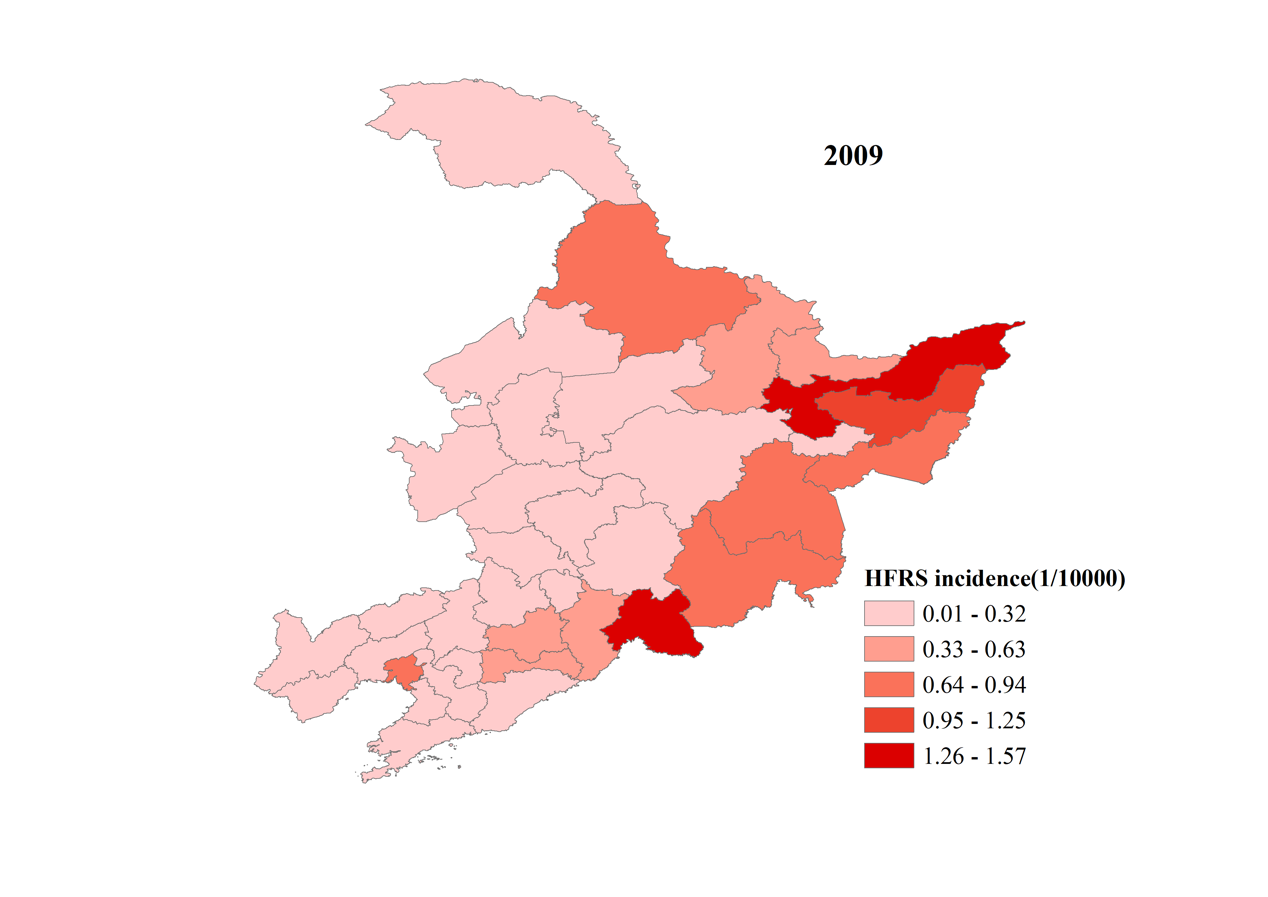


**Figure S5.** Yearly distribution of HFRS incidence in Northeastern China, 2009.


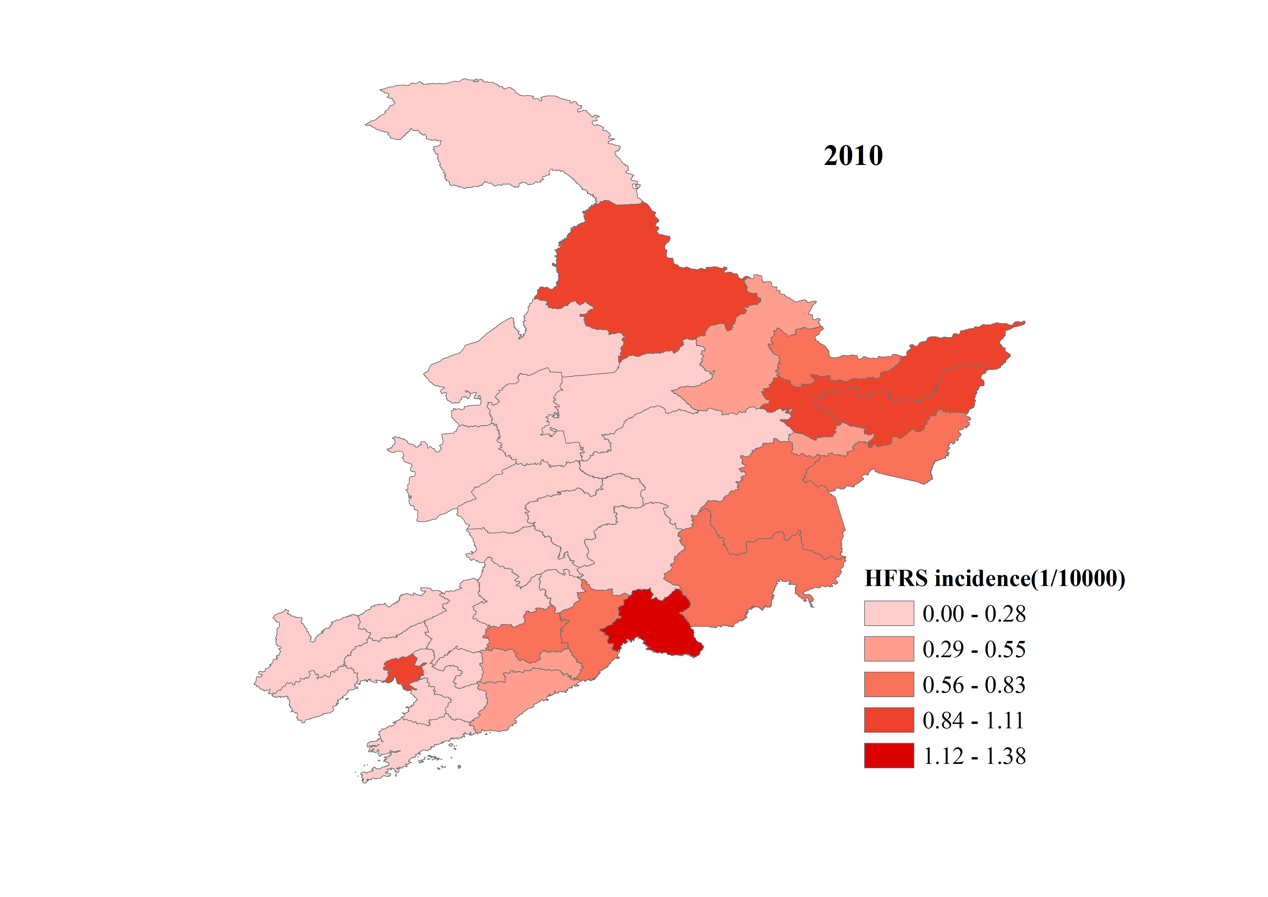


**Figure S6.** Yearly distribution of HFRS incidence in Northeastern China, 2010.


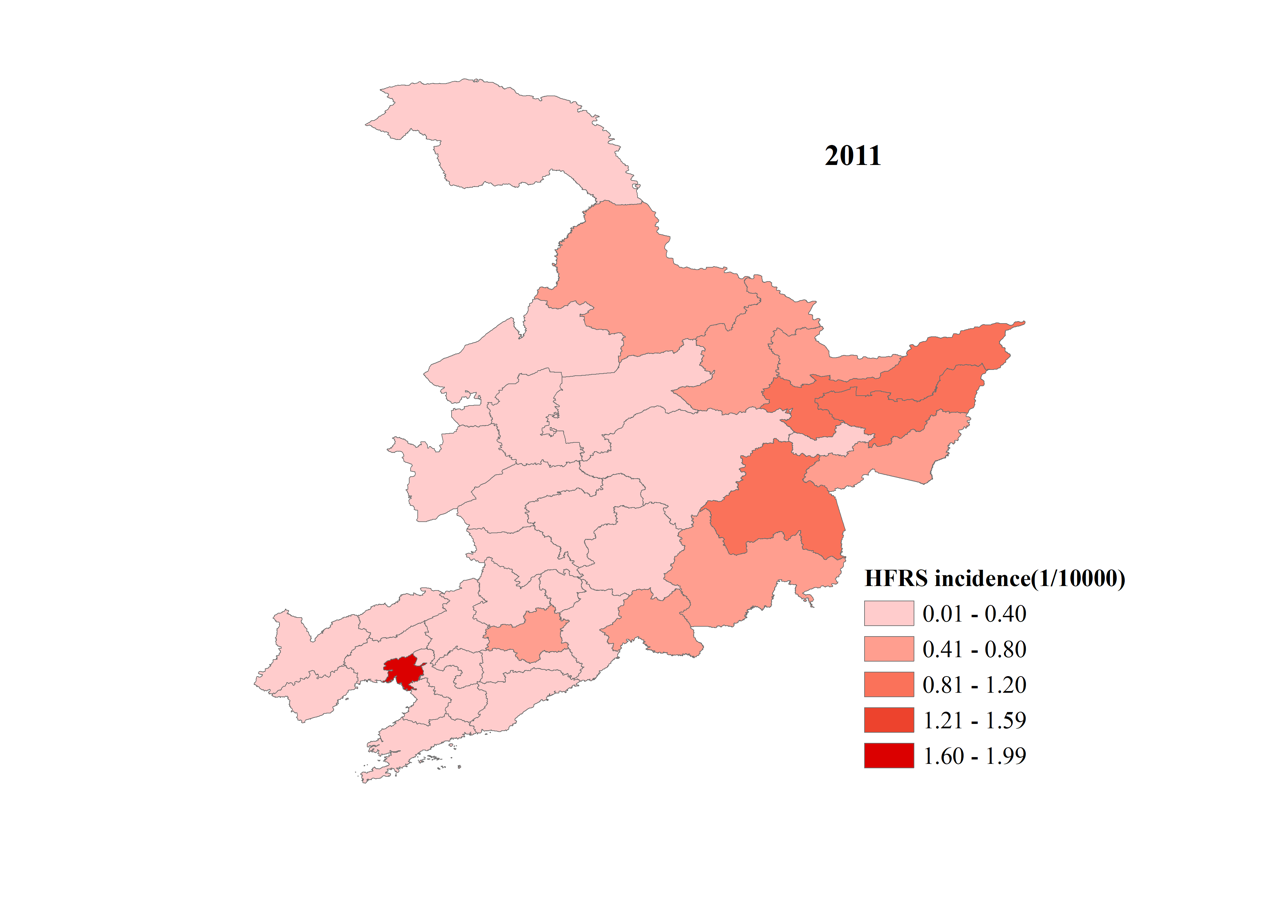


**Figure S7.** Yearly distribution of HFRS incidence in Northeastern China, 2011.

**
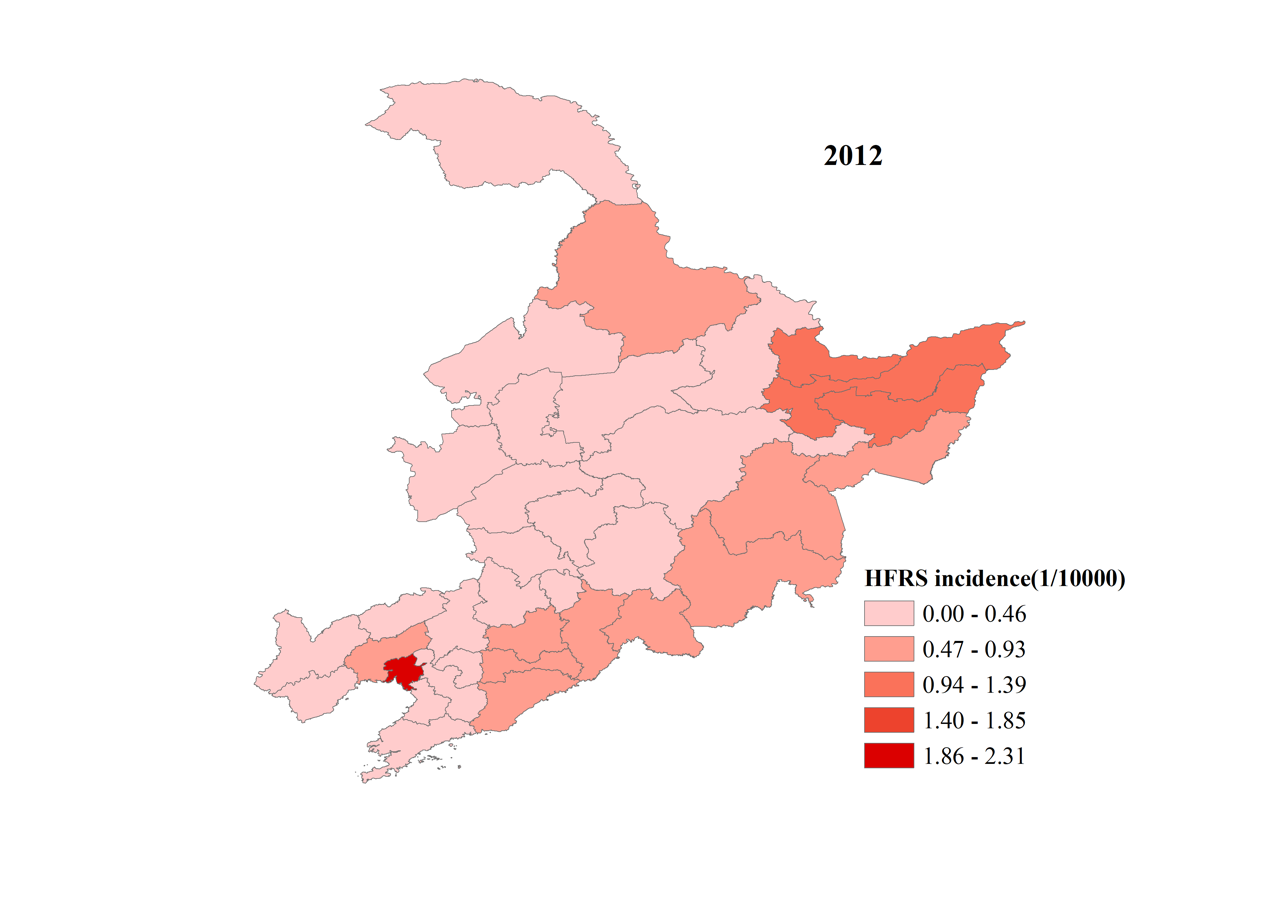
**

**Figure S8.** Yearly distribution of HFRS incidence in Northeastern China, 2012.


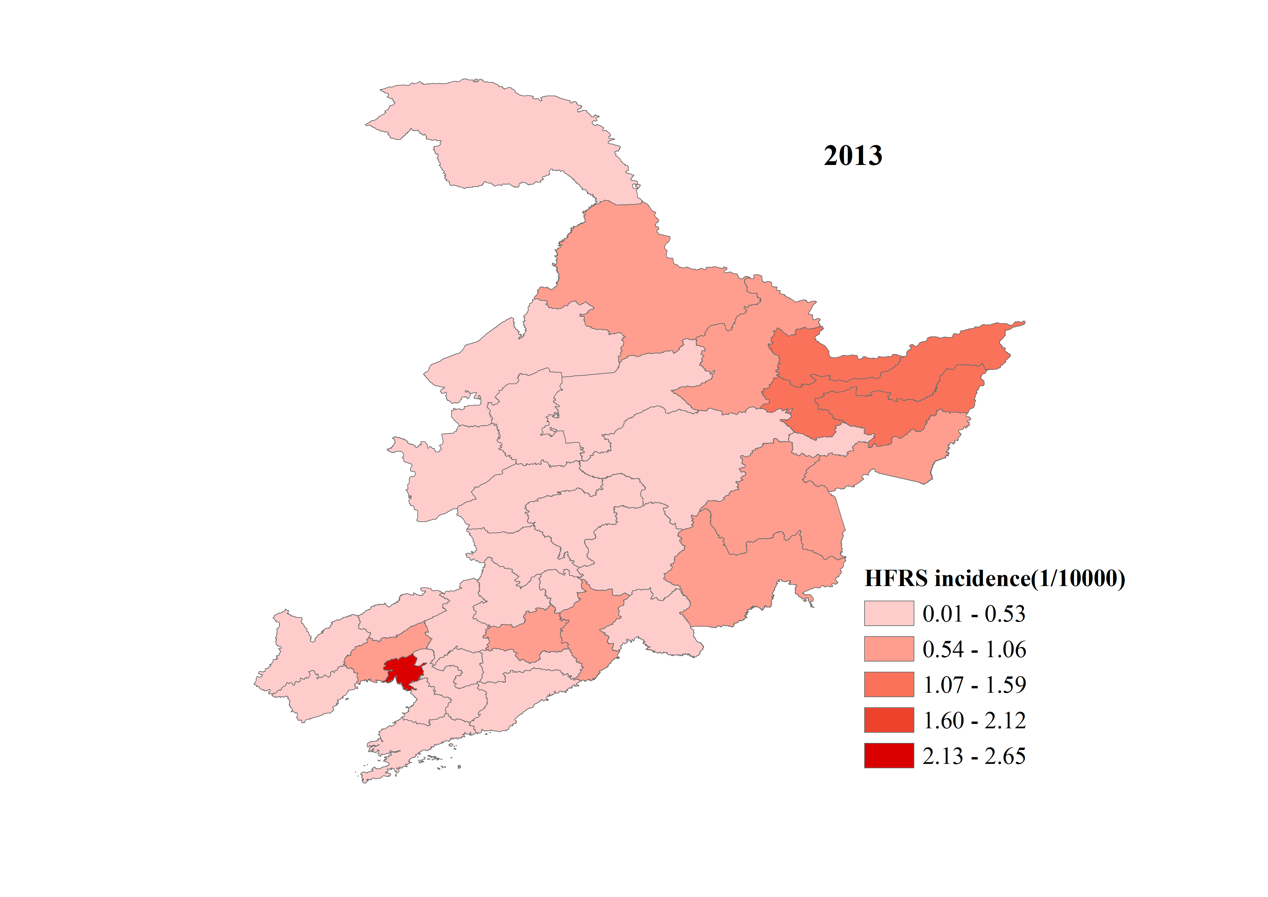


**Figure S9.** Yearly distribution of HFRS incidence in Northeastern China, 2013.

**
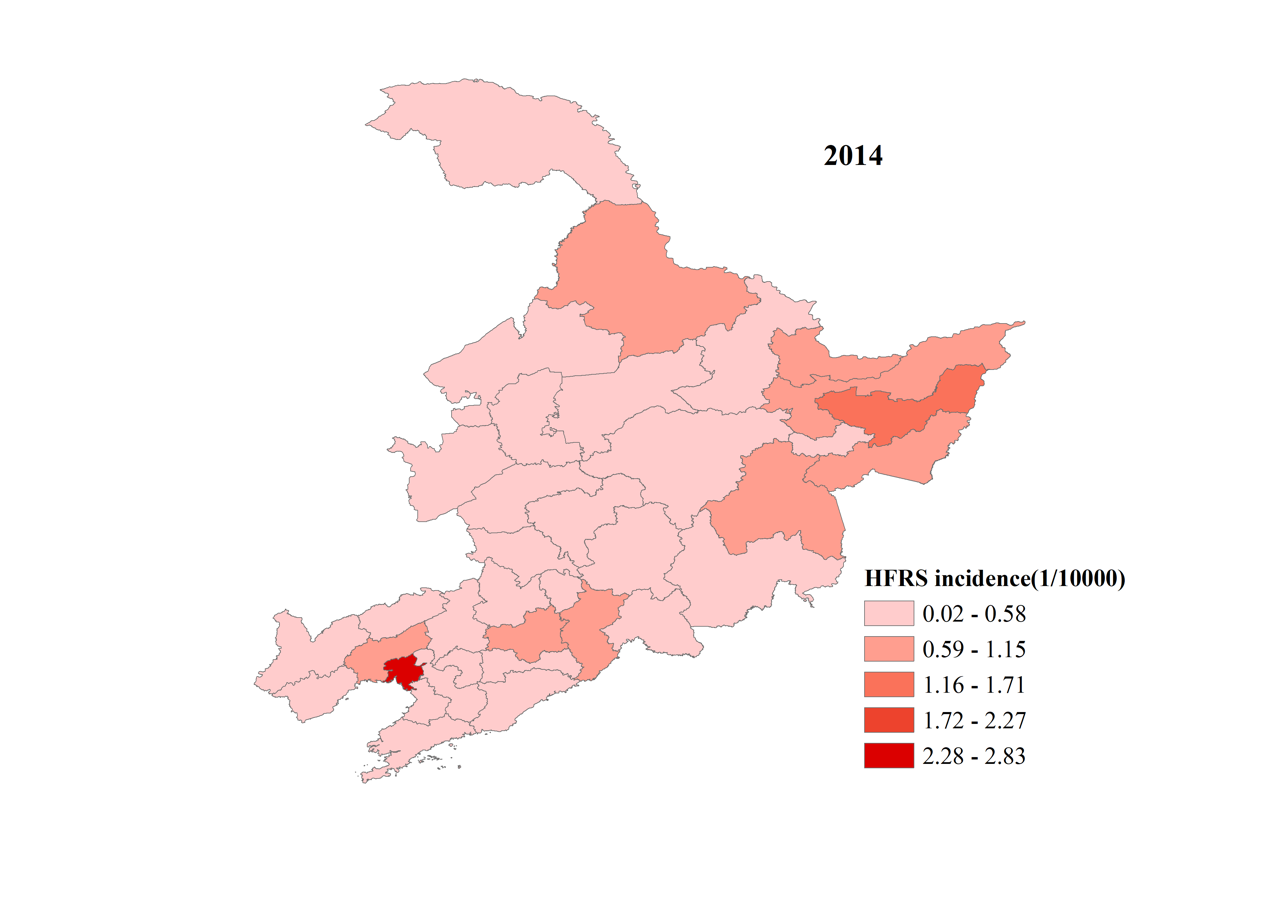
**

**Figure S10.** Yearly distribution of HFRS incidence in Northeastern China, 2014.


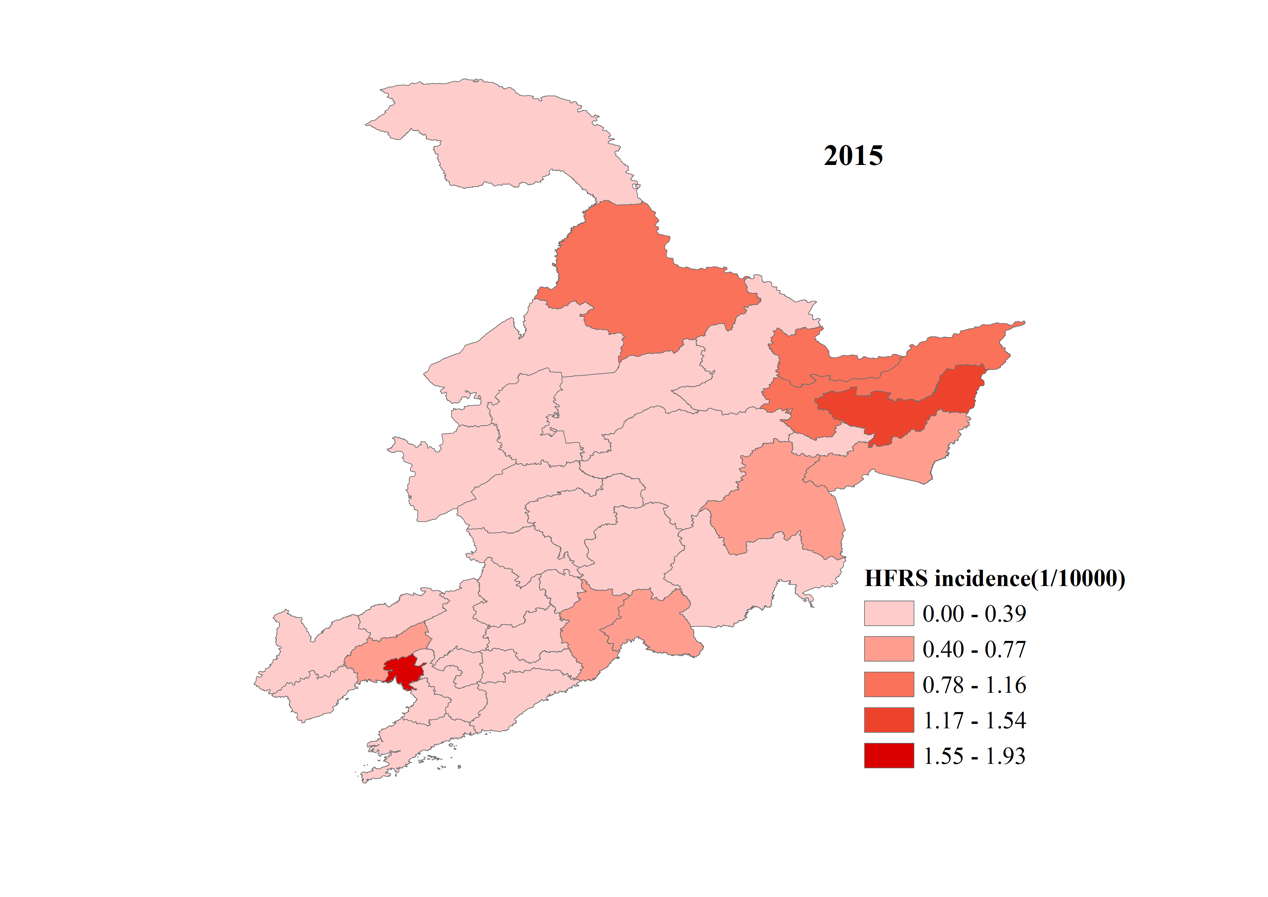


**Figure S11.** Yearly distribution of HFRS incidence in Northeastern China, 2015.

**
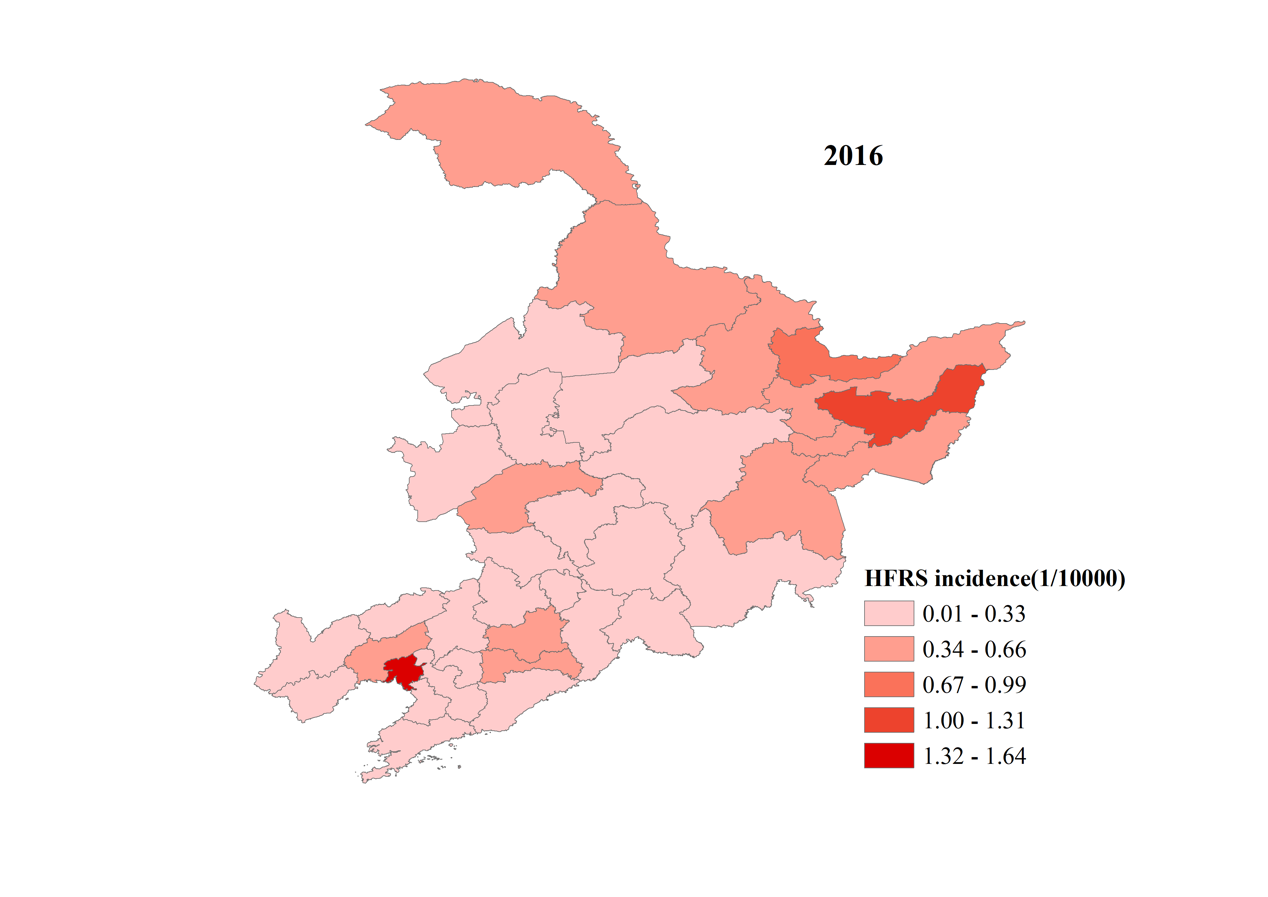
**

**Figure S12.** Yearly distribution of HFRS incidence in Northeastern China, 2016.


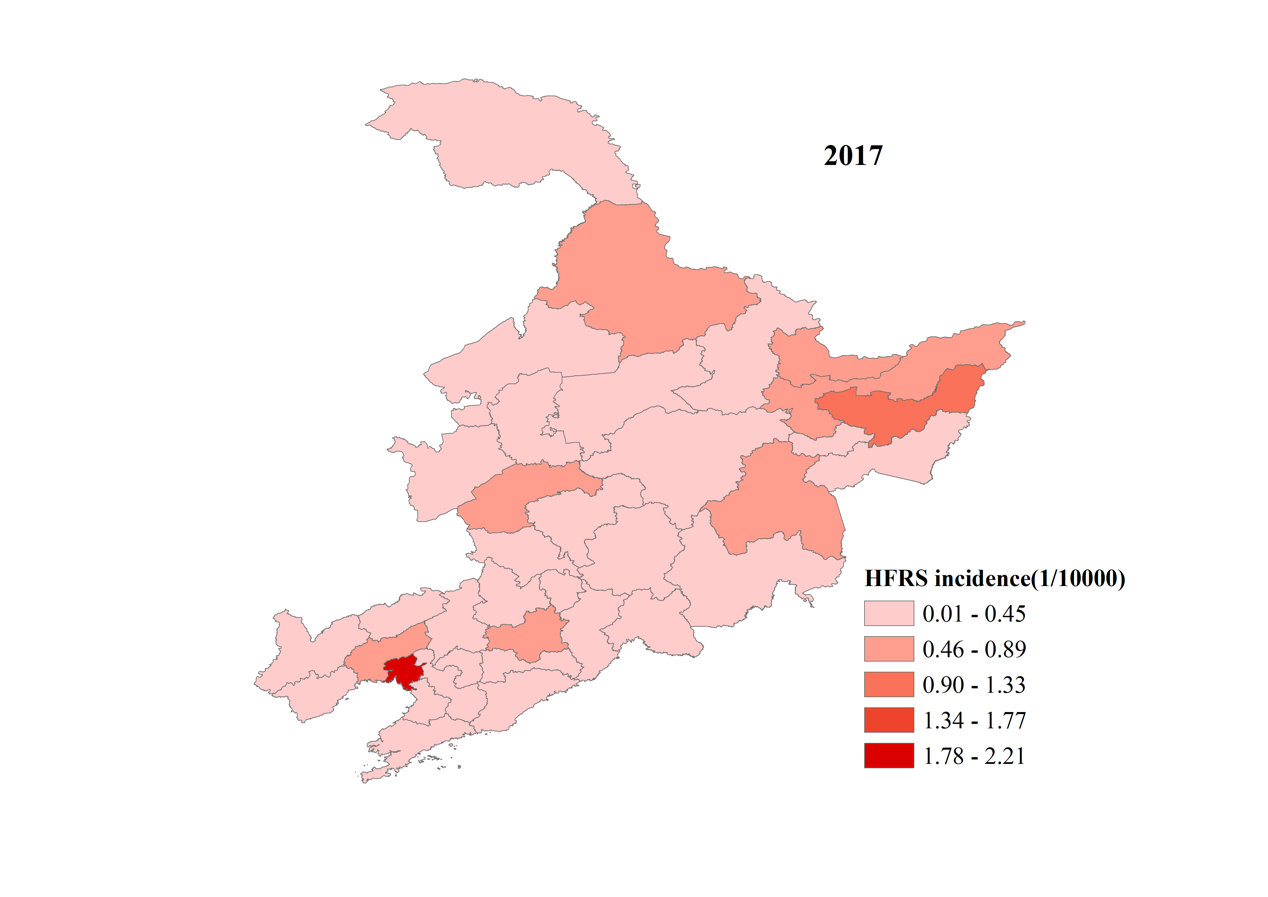


**Figure S13.** Yearly distribution of HFRS incidence in Northeastern China, 2017.

**
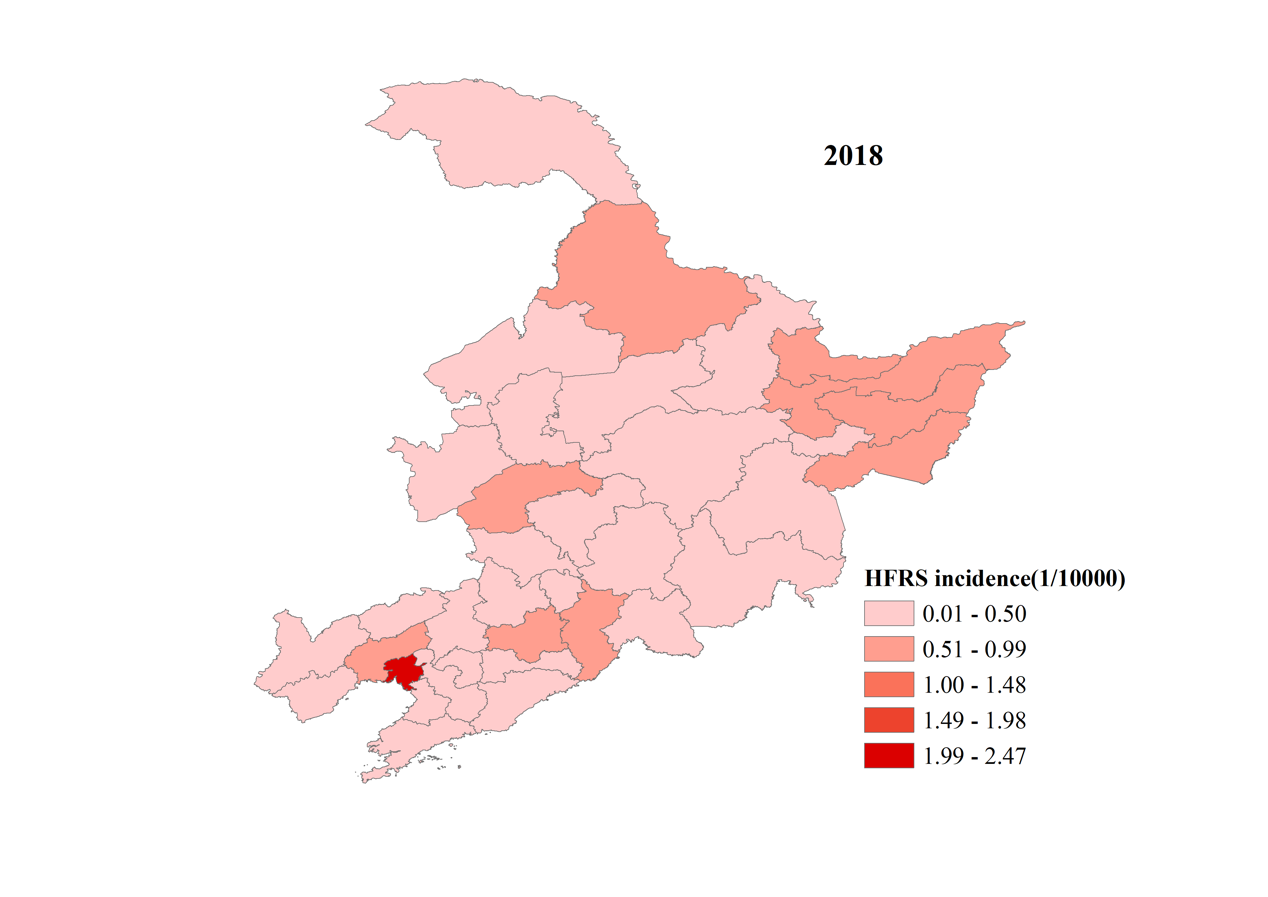
**

**Figure S14.** Yearly distribution of HFRS incidence in Northeastern China, 2018.


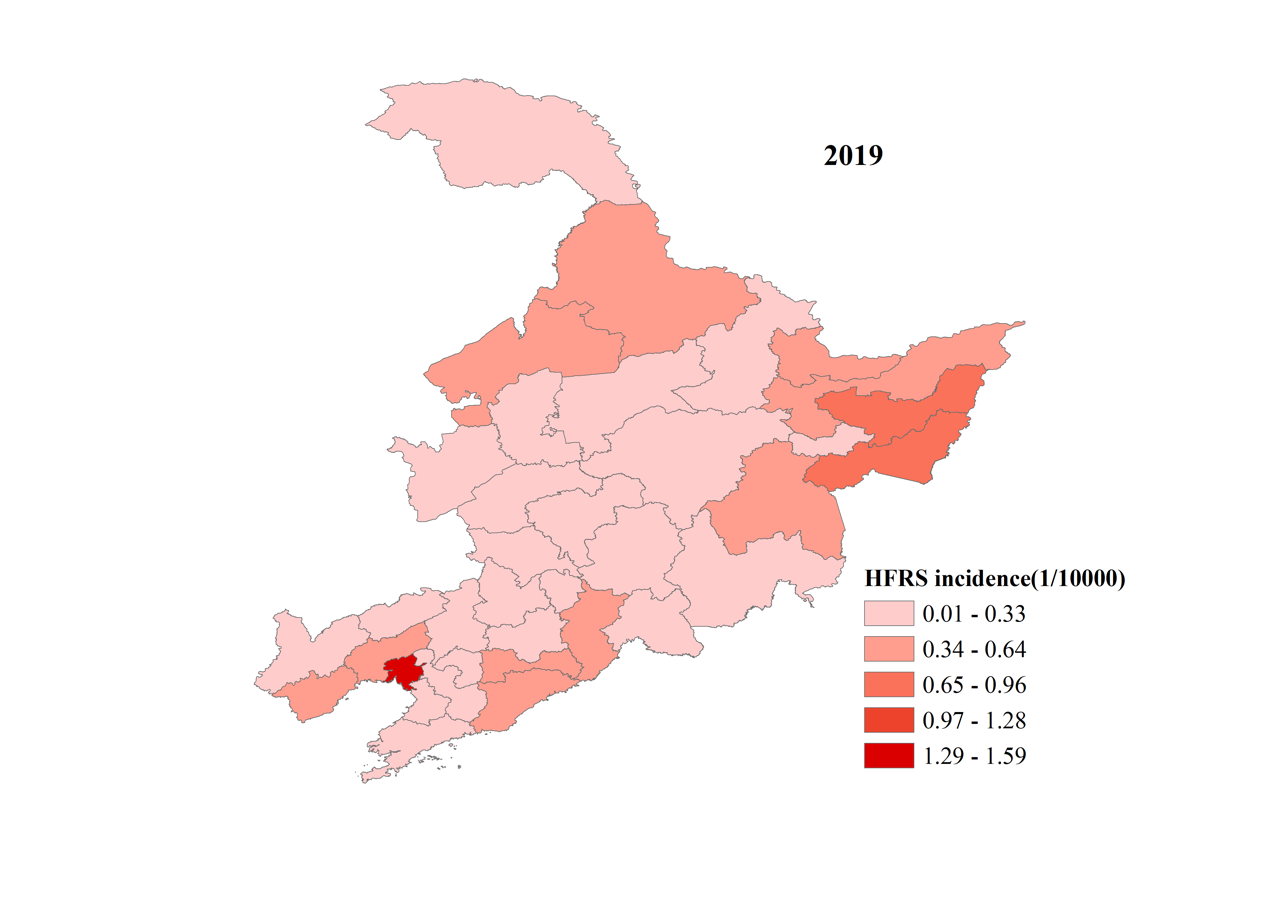


**Figure S15.** Yearly distribution of HFRS incidence in Northeastern China, 2019.

**
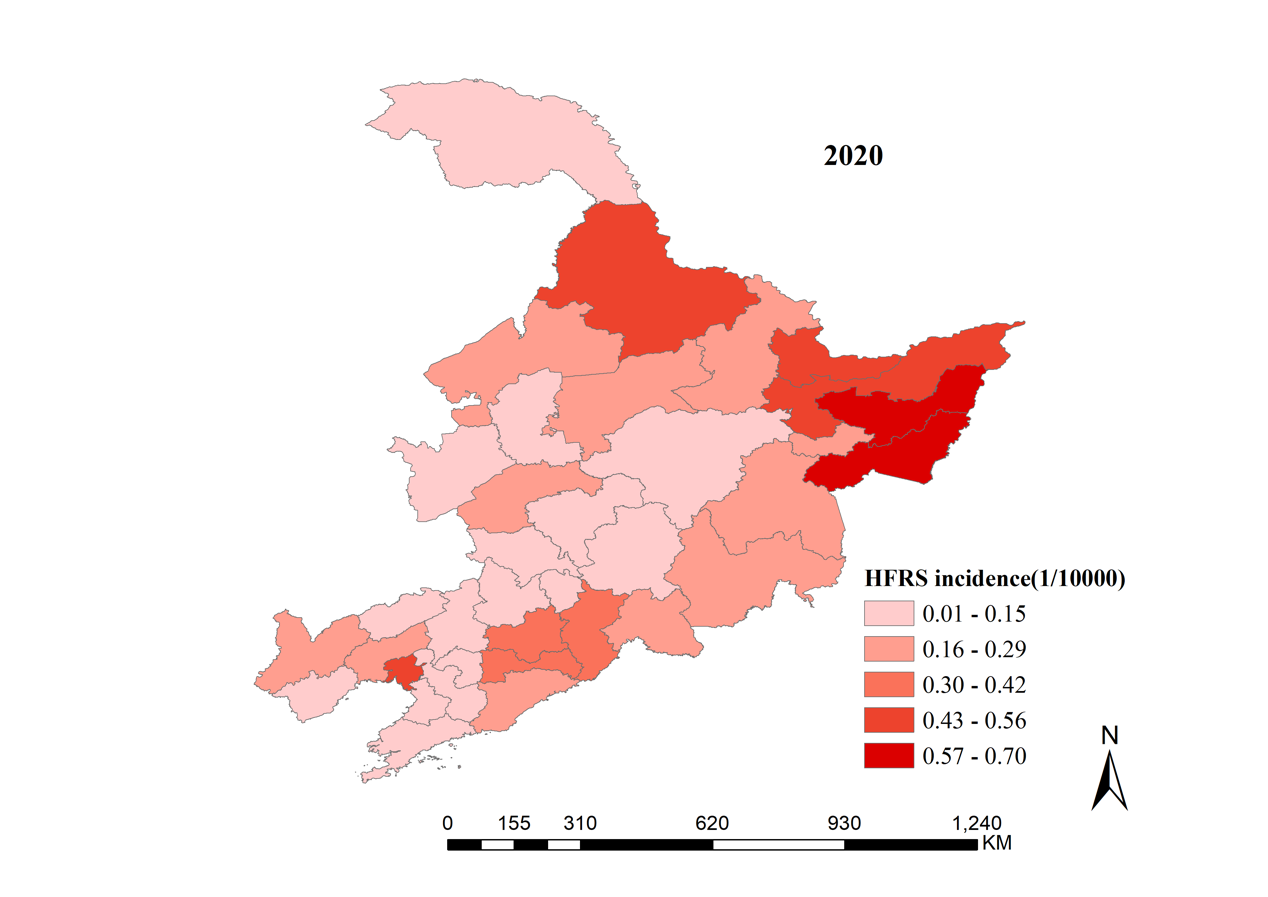
**

**Figure S16.** Yearly distribution of HFRS incidence in Northeastern China, 2020.
