# Supplementary material for: The Spatiotemporal Pattern and Its Determinants of Hemorrhagic Fever With Renal Syndrome in Northeastern China: Spatiotemporal Analysis
Source: JMIR Public Health Surveill. 2023 May 18;9:e42673. doi: 10.2196/42673 (PMC10236282; doi:10.2196/42673)
Supplement: Multimedia Appendix 2 [file publichealth_v9i1e42673_app2.doc]

**Table S1.** The q statistic and p value calculated by factor detector in the Northeastern China, 2006-2020.

| **Province** | **Factor** | **q statistic** | **p value** |
| --- | --- | --- | --- |
| Liaoning Province | WIN_lag4 | 4.94% | ＜0.001 |
|  | MAXEVP_lag3 | 2.90% | ＜0.001 |
|  | MinEVP_lag3 | 2.75% | ＜0.001 |
|  | GST_lag1 | 5.35% | ＜0.001 |
|  | PRE_lag3 | 2.09% | ＜0.001 |
|  | RHU_lag1 | 4.88% | ＜0.001 |
|  | SSD_lag4 | 3.25% | ＜0.001 |
|  | TEM_lag1 | 5.79% | ＜0.001 |
|  | PRS_lag4 | 3.06% | ＜0.001 |
| Jilin Province | WIN_lag6 | 3.13% | ＜0.001 |
|  | MAXEVP_lag5 | 5.36% | ＜0.001 |
|  | MinEVP_lag5 | 4.19% | ＜0.001 |
|  | GST_lag3 | 3.78% | ＜0.001 |
|  | PRE_lag6 | 5.52% | ＜0.001 |
|  | PRS | 5.26% | ＜0.001 |
|  | RHU_lag6 | 3.02% | ＜0.001 |
|  | SSD_lag5 | 4.72% | ＜0.001 |
|  | TEM_lag3 | 3.72% | ＜0.001 |
| Heilongjiang Province | WIN_lag4 | 4.19% | ＜0.001 |
|  | MAXEVP_lag6 | 4.81% | ＜0.001 |
|  | MinEVP_lag2 | 5.34% | ＜0.001 |
|  | GST_lag4 | 14.65% | ＜0.001 |
|  | PRE_lag4 | 7.33% | ＜0.001 |
|  | PRS_lag5 | 7.35% | ＜0.001 |
|  | RHU_lag4 | 5.36% | ＜0.001 |
|  | SSD | 3.91% | ＜0.001 |
|  | TEM_lag4 | 15.49% | ＜0.001 |
| Northeastern China | WIN_lag1 | 1.40% | ＜0.001 |
|  | MAXEVP_lag2 | 1.87% | ＜0.001 |
|  | MinEVP_lag6 | 1.46% | ＜0.001 |
|  | GST_lag1 | 4.18% | ＜0.001 |
|  | PRE_lag3 | 3.54% | ＜0.001 |
|  | PRS_lag5 | 1.23% | ＜0.001 |
|  | RHU_lag4 | 2.13% | ＜0.001 |
|  | SSD | 2.02% | ＜0.001 |
|  | TEM_lag4 | 3.31% | ＜0.001 |

PRS: mean pressure.

lag# refers to the number of months of lag.

WIN: mean wind speed.

MinEVP: minimum evaporation.

MaxEVP: maximum evaporation.

SSD: sunshine duration.

RHU: mean relative humidity.

TEM: mean temperature.

PRE: precipitation.

GST: mean ground temperature.

**Table S2.** The result of interaction detector in the Northeastern China, 2006-2020.

| Northeastern China | GST-lag1 | PRE_lag3 | TEM_lag4 | RHU_lag4 | SSD | PRS_lag4 | MAX_EVP_lag2 | MIN_EVP_lag6 | PRE_lag5 |
| --- | --- | --- | --- | --- | --- | --- | --- | --- | --- |
| GST-lag1 |  |  |  |  |  |  |  |  |  |
| PRE_lag3 | 0.0707 |  |  |  |  |  |  |  |  |
| TEM_lag4 | 0.0755 | 0.0465 |  |  |  |  |  |  |  |
| RHU_lag4 | 0.0967 | 0.0351 | 0.0602 |  |  |  |  |  |  |
| SSD | 0.0826 | 0.0351 | 0.0607 | 0.0367 |  |  |  |  |  |
| PRS_lag4 | 0.1032 | 0.0601 | 0.0780 | 0.0865 | 0.0603 |  |  |  |  |
| MAX_EVP_lag2 | 0.0706 | 0.0489 | 0.0697 | 0.0609 | 0.0460 | 0.0614 |  |  |  |
| MIN_EVP_lag6 | 0.0817 | 0.0426 | 0.0776 | 0.0429 | 0.0454 | 0.0691 | 0.0416 |  |  |
| PRE_lag5 | 0.0626 | 0.0272 | 0.0317 | 0.0174 | 0.0112 | 0.0352 | 0.0270 | 0.0264 |  |
| WIN_lag1 | 0.0957 | 0.0460 | 0.0652 | 0.0878 | 0.0532 | 0.0578 | 0.0458 | 0.0645 | 0.0246 |

PRS: mean pressure.

lag# refers to the number of months of lag.

WIN: mean wind speed.

MinEVP: minimum evaporation.

MaxEVP: maximum evaporation.

SSD: sunshine duration.

RHU: mean relative humidity.

TEM: mean temperature.

PRE: precipitation.

GST: mean ground temperature.

**111**

**Table S3.** The result of interaction detector in Jilin Province, 2006-2020.

| Jilin Province | GST-lag3 | PRE_lag6 | TEM_lag3 | RHU_lag6 | SSD_lag5 | PRS | MAX_EVP_LAG5 | MIN_EVP_lag5 |
| --- | --- | --- | --- | --- | --- | --- | --- | --- |
| GST-lag3 |  |  |  |  |  |  |  |  |
| PRE_lag6 | 0.1113 |  |  |  |  |  |  |  |
| TEM_lag3 | 0.0493 | 0.1048 |  |  |  |  |  |  |
| RHU_lag6 | 0.0729 | 0.0944 | 0.0783 |  |  |  |  |  |
| SSD_lag5 | 0.0895 | 0.1170 | 0.0955 | 0.0759 |  |  |  |  |
| PRS | 0.1178 | 0.1289 | 0.1130 | 0.0887 | 0.0948 |  |  |  |
| MAX_EVP_LAG5 | 0.0862 | 0.1190 | 0.0952 | 0.0844 | 0.0850 | 0.1006 |  |  |
| MIN_EVP_lag5 | 0.1014 | 0.1288 | 0.1114 | 0.0811 | 0.0816 | 0.0918 | 0.0692 |  |
| WIN_lag6 | 0.0713 | 0.0941 | 0.0787 | 0.0549 | 0.0761 | 0.0784 | 0.0921 | 0.0978 |

PRS: mean pressure.

lag# refers to the number of months of lag.

WIN: mean wind speed.

MinEVP: minimum evaporation.

MaxEVP: maximum evaporation.

SSD: sunshine duration.

RHU: mean relative humidity.

TEM: mean temperature.

PRE: precipitation.

GST: mean ground temperature.

**Table S4.** The result of interaction detector in Liaoning Province, 2006-2020

|  | TEM_lag1 | GST_lag1 | WIN_1ag4 | RHU_lag1 | SSD_lag4 | PRS_lag4 | MAX_EVP_lag3 | MIN_EVP_lag3 |
| --- | --- | --- | --- | --- | --- | --- | --- | --- |
| TEM_lag1 |  |  |  |  |  |  |  |  |
| GST_lag1 | 0.0623 |  |  |  |  |  |  |  |
| WIN_1ag4 | 0.0849 | 0.0831 |  |  |  |  |  |  |
| RHU_lag1 | 0.1092 | 0.1068 | 0.0940 |  |  |  |  |  |
| SSD_lag4 | 0.0791 | 0.0744 | 0.0765 | 0.0817 |  |  |  |  |
| PRS_lag4 | 0.1074 | 0.1032 | 0.0961 | 0.0999 | 0.0814 |  |  |  |
| MAX_EVP_lag3 | 0.0755 | 0.0730 | 0.0706 | 0.0699 | 0.0592 | 0.0673 |  |  |
| MIN_EVP_lag3 | 0.0758 | 0.0685 | 0.0787 | 0.0699 | 0.0609 | 0.0707 | 0.0415 |  |
| PRE_lag3 | 0.0749 | 0.0707 | 0.0868 | 0.0885 | 0.0655 | 0.0601 | 0.0521 | 0.0501 |

PRS: mean pressure.

lag# refers to the number of months of lag.

WIN: mean wind speed.

MinEVP: minimum evaporation.

MaxEVP: maximum evaporation.

SSD: sunshine duration.

RHU: mean relative humidity.

TEM: mean temperature.

PRE: precipitation.

GST: mean ground temperature.

**Table S5.** The result of interaction detector in Heilongjiang Province, 2006-2020.

| Heilongjiang Province | GST-lag4 | PRE_lag4 | TEM_lag4 | RHU_lag4 | SSD | PRS_lag5 | MAX_EVP_LAG6 | MIN_EVP_lag2 |
| --- | --- | --- | --- | --- | --- | --- | --- | --- |
| GST-lag4 |  |  |  |  |  |  |  |  |
| PRE_lag4 | 0.2104 |  |  |  |  |  |  |  |
| TEM_lag4 | 0.1715 | 0.1964 |  |  |  |  |  |  |
| RHU_lag4 | 0.1707 | 0.1037 | 0.1722 |  |  |  |  |  |
| SSD | 0.1683 | 0.1085 | 0.1756 | 0.0820 |  |  |  |  |
| PRS_lag5 | 0.2680 | 0.1966 | 0.2545 | 0.1428 | 0.1508 |  |  |  |
| MAX_EVP_LAG6 | 0.1828 | 0.1205 | 0.1822 | 0.0929 | 0.0894 | 0.1612 |  |  |
| MIN_EVP_lag2 | 0.1891 | 0.1387 | 0.2010 | 0.1395 | 0.1051 | 0.1404 | 0.1515 |  |
| WIN_lag4 | 0.1945 | 0.1288 | 0.1887 | 0.0863 | 0.0998 | 0.1439 | 0.0881 | 0.1270 |

PRS: mean pressure.

lag# refers to the number of months of lag.

WIN: mean wind speed.

MinEVP: minimum evaporation.

MaxEVP: maximum evaporation.

SSD: sunshine duration.

RHU: mean relative humidity.

TEM: mean temperature.

PRE: precipitation.

GST: mean ground temperature.

**Table S6.**  Performance of SARIMA model(4, 1, 3)(1, 0, 1)[4] on fitting and forecasting HFRS cases

| Model | MAE | MAPE | RMSE |
| --- | --- | --- | --- |
| Train | 61.89 | 0.17 | 85.27 |
| Test | 56.45 | 0.39 | 72.11 |
| Summary | 60.38 | 0.23 | 81.83 |

The goodness-of-fit was quantified by mean absolute error (MAE), Mean Absolute Percentage Error (MAPE) and root mean square error (RMSE)
